# Insights into preclinical models of calcific aortic valve disease and their translational potential

**DOI:** 10.3389/fcvm.2025.1621990

**Published:** 2025-11-06

**Authors:** Isabelle Lafosse, Romuald Mentaverri, Carine Avondo, Youssef Bennis, Christophe Tribouilloy, Lucie Hénaut

**Affiliations:** 1 UR UPJV 7517 MP3CV, CURS, Amiens, France; 2 Department of Biochemistry and Endocrine Biology, Amiens University Hospital, Amiens, France; 3 Department of Pharmacology, Amiens University Hospital, Amiens, France; 4 Department of Cardiology, Amiens University Hospital, Amiens, France

**Keywords:** aortic valve, calcific aortic valve disease, preclinical models, valvular interstitial cells, translational potential

## Abstract

Calcific aortic valve disease (CAVD) is characterized by a fibrocalcific remodeling of the aortic valve. This pathology is the most prevalent valvular heart disease worldwide and is associated with a poor prognosis. Despite extensive research, no pharmacological treatments are available to slow or reverse valvular degeneration, making aortic valve replacement the only current therapeutic option. This lack of clinical success may stem from an incomplete understanding of the disease's mechanisms and the limitations of current preclinical models, which do not fully replicate the complexity of CAVD and its associated risk factors and comorbidities. Indeed, while existing models offer valuable insights, a deeper understanding of CAVD requires incorporating comorbidities, gender-specific mechanisms, and dynamic cellular and tissue-level changes. This review aims to provide the reader with an overview of preclinical models developed in recent years to study CAVD, assessing their strengths and limitations. We review how these models can be used to mimic and/or investigate the cellular and molecular mechanisms involved in CAVD development, and highlight how key risk factors and comorbidities can be incorporated to enhance the translational potential of research. We hope that this approach will help guide researchers in selecting the most appropriate model for their studies, with the goal of advancing the identification of effective therapeutic candidates.

## 1 Introduction

Calcific aortic valve disease (CAVD) is the most common valvular disease worldwide, affecting approximately 10% of individuals over 65 years old (1, 2). It begins with aortic sclerosis, a mild thickening of the aortic valve (AV) leaflets without significant blood flow obstruction, and may progress to severe calcification, restricting leaflet motion. Over time, this remodeling narrows the AV opening, leading to aortic stenosis (AS), which impedes outflow from the left ventricle, increases afterload, and disrupts hemodynamics. Untreated AS causes left ventricular hypertrophy and dysfunction, resulting in symptoms such as dyspnea, angina, and syncope, ultimately leading to heart failure (HF) and death.

Transthoracic Doppler echocardiography is the standard method for assessing AS severity. Hallmark features include reduced aortic valve area (AVA), increased peak aortic transvalvular velocity, and elevated mean pressure gradient. Based on these parameters, AS is classified as mild, moderate, or severe (3). Computed tomography of aortic valve calcification (CT-AVC) complements echocardiography by enabling quantification and grading of calcification, with sex-specific thresholds for severe AS set at >1,300 Agatston units (AU) in women and >2,000 AU in men (3–5). Once symptomatic, severe AS has a ∼50% two-year mortality if left untreated (6). To date, aortic valve replacement (AVR), either surgical (SAVR) or transcatheter (TAVR), remains the only curative therapy for CAVD (6–8), as no pharmacological treatment can prevent its onset or progression. While preclinical studies have yielded encouraging results, none have translated into clinically effective interventions. This persistent translational gap likely reflects the limitations of current experimental models, which do not fully capture the multifactorial nature of CAVD or its frequent association with comorbidities, thereby hampering the clinical applicability of preclinical findings.

This review provides an overview of the current preclinical models used to study CAVD, emphasizing their strengths, limitations, and ability to replicate key cellular mechanisms, risk factors, and comorbidities. Our goal is to share perspectives that may help researchers select suitable models and enhance the relevance of preclinical findings, ultimately accelerating therapeutic development.

## 2 Pathophysiology of CAVD

### 2.1 Structure of the aortic valve

**Figure 1:**
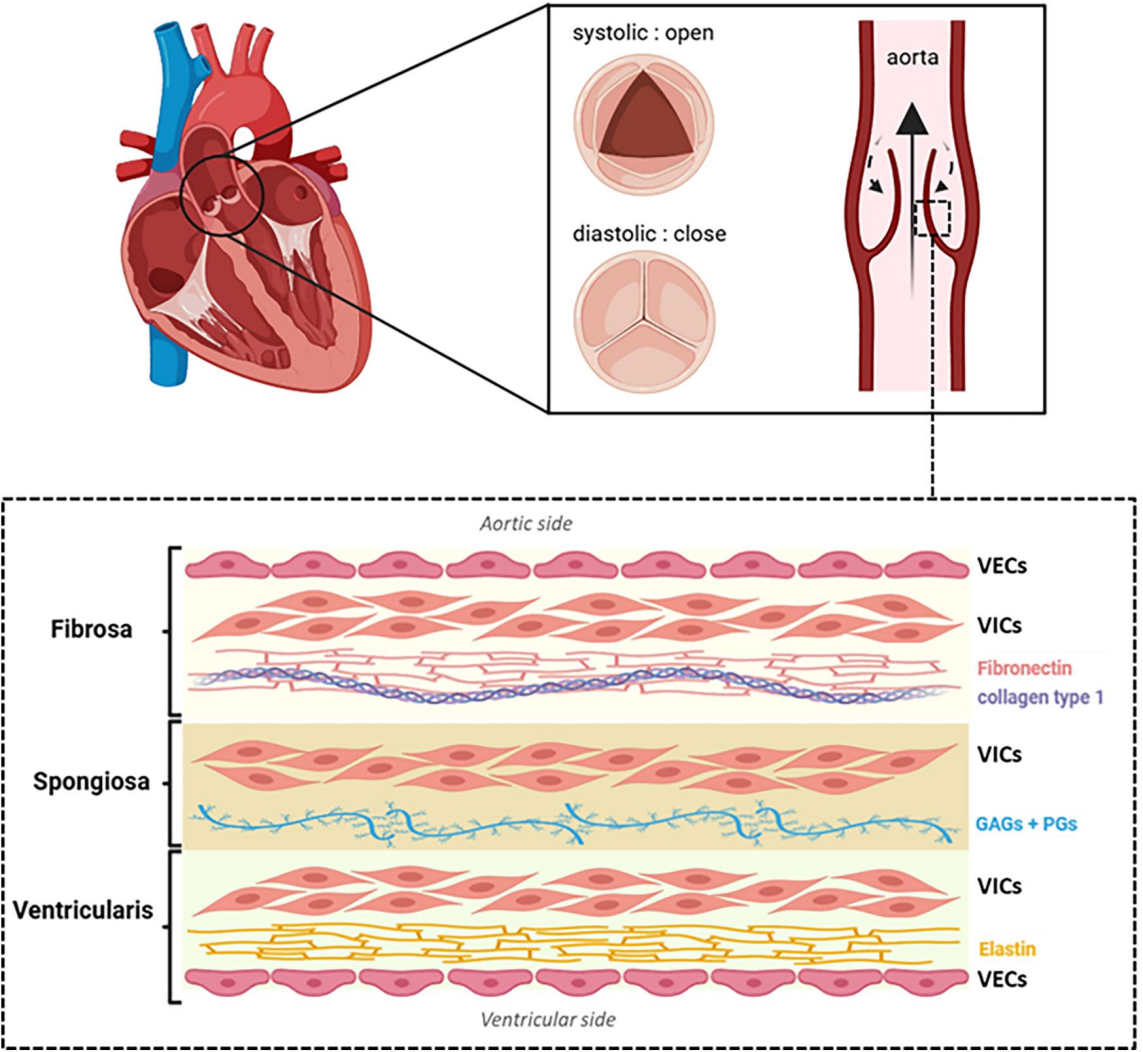
Schematic representation of aortic valve structure. Each leaflet consists of three distinct layers: the fibrosa (rich in fibronectin and collagen type 1 fibers), the spongiosa [composed of glycosaminoglycans [GAGs] and proteoglycans [PGs]] and the ventricularis (rich in elastin). Valvular endothelial cells (VECs) cover both the aortic and ventricular surfaces, while valvular interstitial cells (VICs) are distributed throughout all layers. Illustration created with BioRender.

The healthy AV is an avascular structure, composed of three individual leaflets, located at the junction between the left ventricle and the aorta. Each leaflet contains three distinct extracellular matrix (ECM) layers—fibrosa, spongiosa and ventricularis—lined by valvular endothelial cells (VECs). The fibrosa (∼40% of valve volume), on the aortic side, is rich in fibronectin (FN) and densely packed type I and type III collagen fibrils, providing resistance to mechanical stress and pressure. The spongiosa (∼30% of valve volume), is the central layer. It contains glycosaminoglycans (GAGs), proteoglycans (PGs), and collagen, allowing for shock absorption and efficient cusp opening during systole. The ventricularis (∼20%–30% of valve volume), adjacent to the left ventricle, is enriched in elastin for flexibility and collagen types I, II and III for structural reinforcement (Figure 1). The outer layer of VECs, continuous with the endothelium of the aorta and the left ventricular myocardium, regulates paracrine signaling and exerts antithrombotic effects, maintaining proper valvular function. The three layers are primarily populated by valvular interstitial cells (VICs), whose phenotype and activity are crucial to maintaining structural integrity (9).

### 2.2 Mechanistic insights into CAVD development

#### 2.2.1 Overview of the main mechanisms driving CAVD

**Figure 2:**
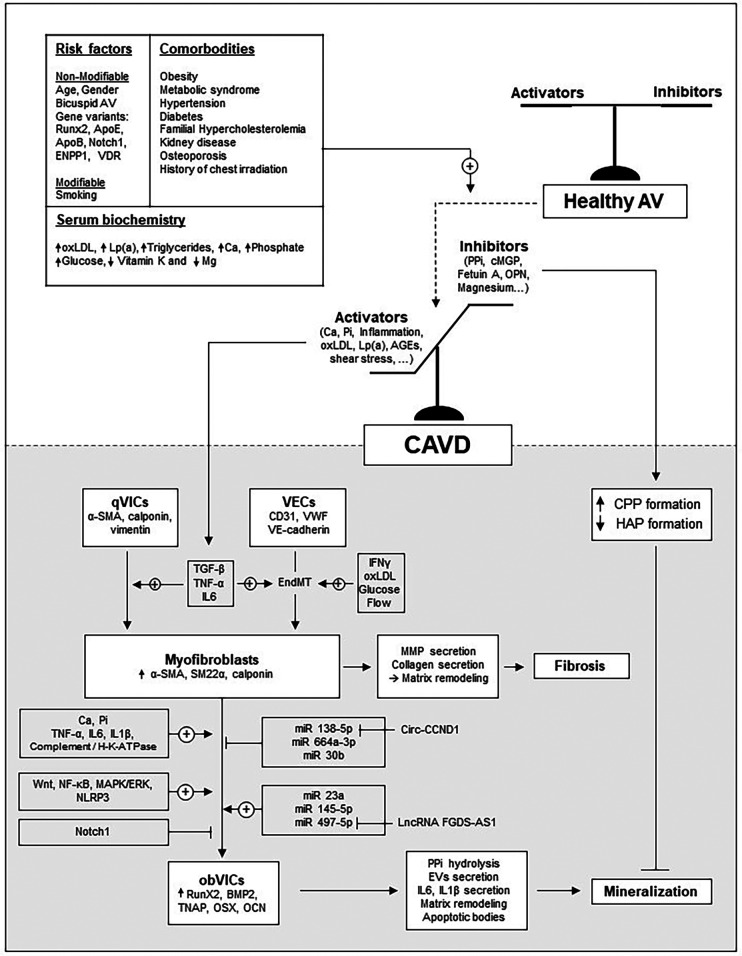
Overview of the main mechanisms driving CAVD development. The schematic illustrates how major risk factors and comorbidities influence the balance between inducers and inhibitors of the mineralization process.

Early CAVD begins with endothelial injury or dysfunction, triggered by mechanical or metabolic stress. This injury promotes lipid deposition and upregulation of adhesion molecules like E-Selectin, VCAM-1 and ICAM-1 on VECs, facilitating immune cell adhesion, rolling and infiltration (10). Cytokines and growth factors released by both immune cells and dysfunctional VECs drive VICs activation (9, 11–13). In particular, TGF-β induces quiescent VICs (qVICs) to differentiate into activated VICs (aVICs), characterized by α-smooth muscle actin (α-SMA) expression and a myofibroblastic phenotype. Inflammation further promotes their proliferation, migration, and secretion of matrix metalloproteinases (MMPs), contributing to leaflet fibrosis, thickening, and stiffening. In early CAVD, elevated TGF-β also promotes the formation of myofibroblasts from VECs via endothelial-to-mesenchymal transition (EndMT) (14, 15). During EndMT, VECs downregulate endothelial markers (e.g., CD31 and VE-Cadherin) and upregulate α-SMA. This process can also be triggered by inflammatory cytokines (IFNγ, IL-6, TNF-α, or LPS) (15), disturbed flow (16), or metabolic factors (oxLDL, HDL, hyperglycaemia) (17). Over time, aVICs and aVECs can transition into osteoblast-like cells (obVICs and obVECs), characterized by decreased α-SMA expression and upregulation of osteogenic markers such as bone morphogenetic protein-2 (BMP2), Runt-related transcription factor-2 (Runx2), and alkaline phosphatase (ALP), thereby promoting mineralization. This cell-mediated fibro-calcific remodeling ultimately stiffens the valve and leads to AS. The main mechanisms driving CAVD are illustrated in Figure 2.

#### 2.2.2 Mechanisms driving mineral deposition

Aortic valve calcification generally reflects an imbalance between inhibitors that prevent calcium-phosphate deposition (mineral phase) and activators that promote VIC/VEC osteogenic transition (cellular phase) (Figure 2).

Among the inhibitors, pyrophosphate (PPi) prevents mineralization by directly interfering with the physicochemical process of hydroxyapatite formation. PPi can be hydrolyzed by ALP, making ALP activity a key regulator of PPi availability. Calcifying VICs show increased ALP activity and decreased PPi levels (18). Other inhibitors include matrix Gla protein (MGP) and fetuin A, which bind calcium and stabilize nascent calcium-phosphate clusters into amorphous, proteinaceous spherical structures known as primary calciprotein particles (CPP), which facilitate calcium-phosphate clearance and prevent ectopic calcification (19–21). MGP expression is significantly reduced in diseased VICs (22) and stenotic AV (23), and circulating Fetuin-A levels are lower in patients with CAVD (24).

On the cellular side, two distinct cell-driven mechanisms are recognized in valve calcification: osteogenic and dystrophic calcification. Osteogenic calcification occurs when VICs and VECs acquire an osteoblast-like phenotype, expressing bone-related markers such as RUNX2, BMP2, and ALP, and producing an osteoid-like ECM that subsequently mineralizes. RUNX2 is a key transcription factor driving osteoblastic commitment by regulating genes such as ALP, osteopontin (OPN), type I collagen, and osteocalcin (OCN). BMP2 promotes osteogenic differentiation by enhancing RUNX2 expression and acetylation, thereby increasing its stability and transcriptional activity. Akin to bone, obVICs release small extracellular vesicles (EVs) enriched in ectonucleotidases that promote calcium-phosphate nucleation (25, 26). Several of these ectonucleotidases, including ALP, are overexpressed in human CAVD samples (27–29). In contrast, dystrophic calcification involves the activation of VICs and VECs into a myofibroblast-like phenotype followed by apoptosis, leading to the formation of apoptotic bodies that closely resemble calcifying EVs and serve as initial nucleation sites for nodule formation. This apoptosis-dependent process is strongly influenced by the mechanical stiffness and ECM composition of the microenvironment, which modulate cytoskeletal tension and thereby the extent of calcification.

Over the years, multiple signaling pathways have been identified as regulators of VIC osteogenic reprogramming. NF-κB signaling induces BMP2 (30) and RUNX2 (31) expression in VICs. This effect involves NF-κB–mediated upregulation of TERT, which binds STAT5 to activate the RUNX2 promoter and drive osteogenic reprogramming (32). The MAPK/ERK pathway also contributes to RUNX2 induction, either through TNF-α stimulation (33, 34) or via complement crosstalk with H-K-ATPase (35, 36). Similarly, NLRP3 activation promotes RUNX2 and ALP expression in VICs (37). Wnt signaling enhances both RUNX2 expression and ALP activity (38), and several of its key components—including the receptor LRP5, the agonist WNT3a, and the nuclear effector β-catenin—are overexpressed in CAVD samples (39). By contrast, activation of Notch1 suppresses BMP2 and RUNX2 expression in cultured VICs (40, 41), consistent with the inverse correlation between Notch1 and RUNX2 observed in CAVD samples (42).

In addition to these signaling pathways, recent studies have demonstrated that dysregulation of miRNAs also contributes to the osteogenic reprogramming of VICs in AS (43). For instance, miR-664a-3p, which is downregulated in mineralized AV, inhibits VICs osteogenic differentiation by directly binding to BMP2 and repressing its expression (44). MiR-30b, which expression is also downregulated in mineralized AV, suppresses VICs osteogenic differentiation by directly inhibiting RUNX2 expression (45, 46). Conversely, miR-23a, which is upregulated in mineralized AV, promotes VIC mineralization by enhancing RUNX2 activation through the suppression of Notch1 expression (42). Recently, Goody et al. observed that miR-145-5p is one of the most strongly upregulated miRNAs in CAVD, with its vesicular content increased in the disease. *In vitro* calcification experiments demonstrated that EV-mediated transfer of miR-145-5p promotes ALP expression by suppressing ZEB2, a negative regulator of the ALPL gene (47). Interestingly, circ-CCND1, whose expression is upregulated in CAVD samples, was shown to promote osteogenic transition of VICs by sponging miR-138-5p, thereby activating the osteogenic CCND1/P53/P21 pathway (48). By contrast, LncRNA FGD5-AS1, which is downregulated in CAVD patients, sponges miR-497-5p to regulate BIRC5, thereby repressing osteogenic differentiation and alleviating CAVD (49). The main signaling pathways involved in VIC phenotypic changes are summarized in Table 1.

**Table 1:** Main markers used to identify the phenotypic states of VICs and VECs.

Markers for VECs			Markers for VICs		ECM composition		
Healthy VECs	aVECs (EndMT)	obVEC	aVICs	obVICs	Fibrosa	Spongiosa	Ventricularis
vWF+ CD31+ VE-Cadherin+ CD105+ αSMA- SM22-α- Calponin- Vimentin-	↓ vWF ↓ CD31 ↓ VE-Cadherin ↓ CD105 ↑ αSMA ↑ SM22-α ↑ Calponin ↑ Vimentin	↓ αSMA ↓ SM22-α ↓ Calponin ↓ Vimentin ↑ RUNX2 ↑ BMP2 ↑ OSX ↑ OCN ↑ ALP ↑ OPN	vWF- CD31- VE-Cadherin- CD105- αSMA+ SM22-α+ Calponin+ Vimentin+	↓ αSMA ↓ SM22-α ↓ Calponin ↓ Vimentin ↑ RUNX2 ↑ BMP2 ↑ OSX ↑ OCN ↑ ALP ↑ OPN	Fibronectin Collagen I Collagen III	Glycosaminoglycans (hyaluronic acid, chondroitin sulfate) Proteoglycans (versican, decorin)	Elastin Collagen I Collagen II Collagen III

### 2.3 Risk factors and comorbidities

Older age is the strongest risk factors for AS. Indeed, severe AS affects approximately 3.4% of individuals aged 75 years and older, with symptoms present in 75% of cases (1, 2). Bicuspid aortic valve (BAV), which is a common congenital defect in which the AV has two leaflets rather than three, represents a significant risk factors for AS. Patients with BAV typically develop AS 10–20 years earlier than those with a tricuspid AV, and their lifetime risk of developing the disease is approximately 50%. Although the prevalence of BAV in the general population is estimated at 0.5%–1.0%, it accounts for nearly half of all AVs surgically replaced due to AS in the United States (50). The increased susceptibility to calcification observed in BAVs can be attributed to a combination of hemodynamic and structural factors. Abnormal, turbulent, and asymmetric blood flow generates altered shear stress patterns that promote VIC activation. In parallel, structural abnormalities such as thickened or disorganized valve leaflets and the presence of a raphe—an incomplete separation between cusps—further predispose BAVs to pathological calcification.

Additional clinical factors associated with AS largely overlap with atherosclerosis risk and include male sex, smoking, and metabolic syndrome (e.g., hypercholesterolemia, hypertension, obesity, and diabetes).

Indeed, the risk of AS is twice higher in men compared to women (51, 52). Moreover, men with AS tend to exhibit greater calcification (53), whereas women show increased fibrosis despite similar disease severity (54), highlighting the importance of considering sex as a biological variable in preclinical research. Oxidized LDL (OxLDL) levels correlate with AV inflammation and fibro-calcific remodelling (55, 56), and recent studies implicate the LPA gene, encoding apolipoprotein(a), in AS pathogenesis (57–59). Besides, elevated lipoprotein(a) [Lp(a)] and oxidized phospholipids (Ox-PL) are independently associated with faster AS progression (60). Secondary hyperparathyroidism and renal failure are also associated with AS progression (61).

## 3 *In vitro* modeling of CAVD

To investigate the mechanisms driving CAVD, researchers commonly use *in vitro* models based on VICs and VECs from various species. These cells can be cultured independently or in co-culture, in 2D or 3D systems. The selection of cell type and culture conditions should be carefully tailored to each study's objectives to ensure relevant and reproducible results.

### 3.1 Main sources of VICs/VECs for *in vitro* studies

#### 3.1.1 Human VICs/VECs

##### 3.1.1.1 Main sources and challenges

Most primary VICs and VECs are isolated from AVs of patients undergoing SAVR. However, these tissues are often extensively remodeled, and the resident cells already display features of the disease. To better investigate early mechanisms of AS, valvular cells can instead be isolated from AVs of patients with idiopathic dilated cardiomyopathy or post-infarction heart failure, as well as from non-transplantable donor hearts or accident victims. When working with such primary cultures, it is important to keep in mind that variations in sex, age, and clinical background, along with donor-specific genetic and epigenetic factors, can lead to substantial heterogeneity in cellular responses. While often viewed as a limitation, this variability accurately reflects population diversity and thus enhances the physiological relevance and translational value of *in vitro* findings. In such contexts, access to key donor information (such as sex, age, and comorbidities) is particularly valuable for conducting comparative studies (e.g., male vs. female, young vs. old, tricuspid vs. bicuspid, or diabetic vs. non-diabetic).

##### 3.1.1.2 hVICs/hVECs isolation and culture

Valve retrieval should ideally be performed rapidly to preserve cell viability. Leaflets should be placed immediately in cold saline, PBS, or DMEM, stored at 4 °C, and processed within 12 h. Interestingly, Cuevas et al. demonstrated that, if valve samples are stored in a cold storage solution suitable for organ transplantation, viable VECs and VICs can still be obtained from leaflets processed between 24 and 61 h post-extraction. This information is particularly relevant for centers that do not have immediate access to the medical facilities where valve excision is performed (62).

Although there is some variability in the methods used by researchers to isolate valvular cells, the general approach typically involves a brief enzymatic digestion to remove human VECs (hVECs), followed by a longer digestion to release human VICs (hVICs) (62–66). Most protocols begin with a 10-min incubation of valve tissue in collagenase at 37 °C. The hVECs are then collected by gentle scraping or vortexing, followed by centrifugation and filtration before being seeded onto FN-coated T25 flasks (64, 65). In culture, VECs form rosette-like colonies, reach confluence in about a week, and are passaged at a 1:3 ratio. For VIC isolation, the remaining valve fragments are rinsed with saline, cut into ∼2 mm^2^ pieces, and incubated with collagenase under gentle agitation. Two main protocol variations are described in the literature: short incubations (45 min to 3 h) with high collagenase concentrations (∼1,000 U/ml) (66–68), and longer or overnight digestions with lower concentrations (250–600 U/ml) (64, 65, 69). The strained cell suspension is then centrifuged, and seeded at 800,000 cells per 75 cm^2^ flask. VICs are typically cultured in DMEM supplemented with 10%–15% FBS and antibiotics, displaying a fibroblast-like morphology after 24 h. They are passaged at 90%–95% confluence and seeded at a 1:2 ratio, with medium changes twice weekly. According to Ground et al., successful hVIC culture is more likely when starting with a sufficient amount of tissue, typically greater than 500 mg, and when the initial digestion yields a substantial number of cells, generally exceeding 100,000. They also demonstrated that patient pathology is not predictive of cell culture success, and that a seeding density of 10,000 cells/cm^2^ is ideal for experiments lasting less than 5 days (66).

The purity of cultured cells is a key concern in valvular cell studies, given its impact on experimental outcomes. Cell purity is usually assessed using immunohistochemistry or flow cytometry. VECs should be αSMA-negative and positive for endothelial markers [CD31, VE-cadherin, von Willebrand factor (vWf)], while VICs should show the opposite profile. The presence of αSMA-positive cells in VEC cultures indicates VIC contamination, and double-positive cells (endothelial markers and αSMA) suggests EndMT. To ensure purity, freshly isolated VECs may be labeled with anti-CD31 or anti-CD105 magnetic beads and sorted via Magnetic-Activated Cell Sorting, before first seeding (54, 55). The presence of endothelial markers in VIC cultures suggests VEC contamination. Additional markers like calponin (myofibroblast) and vimentin (mesenchymal stem cell) may help refine phenotypic identification (64, 65, 70, 71). Over successive passages, VICs tend to differentiate into myofibroblasts, characterized by an increased expression of αSMA (70), a process likely driven by the stiffness of plastic culture surfaces (72). Therefore, experiments on VICs are best conducted between passages 2 and 6. According to Ground et al., optimizing the VIC isolation protocol using 1,000 U/ml collagenase for 2 h resulted in the highest number of viable VICs while minimizing aberrant aVIC differentiation (66). The main markers used to identify the different phenotypic states of VICs and VECs are summarized in Table 1.

Until recently, most VICs used in research were derived from primary cultures. However, in 2025, Wang et al. established and characterized a novel immortalized hVIC line (73). They achieved immortalization of primary VICs through lentiviral transduction with SV40 large T antigen (pGMLV-SV40T-PURO), followed by puromycin selection to establish stable cell lines. Compared with primary VICs, these immortalized cells showed higher viability, reduced senescence, stable transcriptomic profiles across multiple passages, and preserved responsiveness to several osteogenic inducers. RNA-seq analyses further revealed the central role of inflammation-related pathways in driving their osteogenic transformation, suggesting that this model may be ideally suited to investigate the contribution of inflammation to VIC phenotypic changes. In the future, this immortalized VIC line could become a valuable and standardized *in vitro* tool for studying AV calcification, particularly for laboratories without easy access to surgical AV specimens.

#### 3.1.2 VICs/VECs from other species

Cells from porcine AVs, which closely resemble human AVs in structure and composition, are a valuable alternative to human cells. Being free of disease, porcine AVs provide an interesting source of cells for studying early CAVD events. Moreover, swine naturally develop atherosclerotic valve lesions, mirroring early human valvular calcification (74). Their large size allows for efficient enzymatic digestion and isolation of pure cell populations. Porcine AVs can be sourced from slaughterhouses, where swine hearts are typically discarded, aligning with the ethical principle of reduction in animal experimentation. Porcine VICs and VECs (pVICs/pVECs) can be isolated using the same protocol as for human cells (64). While porcine and human valvular cells share similarities, there are also notable differences between them (64). For instance, vimentin expression is higher in pVECs than in pVICs, which is the opposite of what is seen in humans. Compared to hVECs, pVECs also show reduced migratory capacity. Additionally, while pVECs respond to high doses of TNF-α by undergoing myofibroblastic transition (15), low doses—effective in hVECs—fail to induce α-SMA and vimentin expression in pVECs (64). Similarly, TGF-β, which induces α-SMA in hVECs, has no such effect in pVECs (75). These interspecies differences, along with the limited availability of molecular tools for the porcine models, should be carefully considered when using swine-derived cells.

In recent years, rodent AVs have gained interest as a source of VICs due to several advantages, including low cost, ease of handling, short lifespan, genetic manipulability of rodents, and access to extensive molecular tools. However, isolation of VICs and VECs from rodents is not yet a gold standard, likely due to the technical challenges associated with working on small tissue samples. Lin et al. showed that VICs can be isolated from rat AV using careful valve dissection followed by enzymatic digestion with collagenase II (76). However, due to the small size of rat AV, approximately 30 leaflets were required to seed a T25 flask, raising ethical concerns. To address this, the authors developed immortalized rat VIC (RAVIC) cell lines via lentiviral transduction with Simian virus (SV40) large T antigen (77). This model enables mechanistic studies of CAVD, which can later be validated in primary cultures to reduce animal use. Primary RAVICs can also be obtained via explant culture, where cusps stripped of endothelium are incubated in growth medium until VICs migrate out (78, 79). To date, few studies have been conducted on RAVICs, so that their phenotypic similarity to human cells remains difficult to evaluate. More recently, VICs have been successfully isolated from mice (80), with only 3 AVs (9 leaflets) required to initiate the culture, opening the door to studies using VICs from genetically modified models. However, rodent valves differ markedly from those of humans and pigs. Their cusps are only ∼5–10 cells thick and lack the distinct trilaminar architecture seen in larger animals (81–83). Moreover, wild-type rodents on standard diets do not develop age-related AV calcification (81), suggesting that AV remodeling mechanisms in mice and rats may not fully replicate those in humans. Further investigation is needed to clarify these interspecies differences.

In 2022, Tao et al. successfully isolated rabbit VICs and used them to investigate how ox-LDL promotes their osteogenic transformation (84). Rabbits are easy to raise, have a moderate lifespan, and their size and anatomy make them suitable for short-term, cost-effective experiments. Despite these advantages, rabbit VICs remain underused in *in vitro* studies. Early studies also used VICs from sheep (85) and dogs (86), but these models have largely been abandoned, mostly due to limited accessibility and ethical concerns.

### 3.2 Inducing phenotypic switching of VICs and VECs *in vitro*

#### 3.2.1 Mimicking VIC myofibrobastic transition

*In vitro*, VIC myofibroblastic differentiation is typically assessed by monitoring the gradual upregulation of α-SMA, calponin, and SM22 (87–89). While TGFβ-1 is the most commonly used inducer of myofibroblastic differentiation *in vitro* (90–92), the response to TGFβ-1 varies across species. Indeed, α-SMA expression increases within 24 h in ovine VICs (93), after 4 days in hVICs (13), and up to 5 days in pVICs (94). In 2004, Walker et al. showed that culturing pVICs at high density and treating them with TGF-β1 induced a myofibroblast-like phenotype and promoted the formation of multicellular aggregates (90). Apoptosis occurred in the central region of these aggregates, where calcium deposits subsequently formed. Fisher et al. later demonstrated that combining mechanical stretch with TGF-β1 rapidly produced pVIC aggregates; central apoptosis again preceded the formation of calcific nodules (95). Inhibition of apoptosis using Z-VAD, a pan-caspase inhibitor that irreversibly blocks caspase activity, markedly reduced the number of calcific nodules, confirming the essential role of apoptosis in initiating dystrophic calcification.

#### 3.2.2 Mimicking VIC osteogenic transition and mineralization

Osteogenic differentiation and mineralization are induced *in vitro* by culturing cells in a medium typically supplemented with β-glycerophosphate (β-GP) and/or elevated calcium and inorganic phosphate (Pi) levels. This medium is commonly referred to as osteogenic medium (OM).

β-GP has been widely used because it can be hydrolyzed by ALP expressed by VICs, releasing Pi that promotes the expression of osteogenic markers such as RUNX2, ALP, OPN, and OCN, as well as mineralization. Its efficacy depends on several factors, including concentration (typically 10 mM), calcium availability in the medium, and exposure time (usually around 21 days to achieve significant mineralization in DMEM containing 1.8 mM calcium). Osteogenic media using β-GP often include dexamethasone (100 nM) to further promote osteogenic differentiation, and ascorbic acid (50 µg/ml) to support collagen synthesis (9). However, because ALP activity is crucial for β-GP hydrolysis, this method may be ineffective in VICs with low ALP expression. To further enhance mineralization, β-GP-based OM can be supplemented with cytokines such as BMP-2, BMP4, BMP7, TGFβ-1 or TGFβ-3, which boost ALP expression (96, 97).

Alternatively, mineralization can be induced by directly increasing calcium and/or Pi concentrations in the culture medium. Calcium-enriched media typically use calcium chloride (CaCl_2_) at 2.5–5 mM (98), while Pi-enriched media rely on sodium phosphate (Na_2_HPO_4_ and/or NaH_2_PO_4_) at 2–5 mM (99, 100) to mimic hyperphosphatemia, as seen in chronic kidney disease (CKD). In this type of system, mineralization becomes detectable within 10–14 days. Combining both ions accelerates the mineralization process, provided their concentrations remain below their solubility threshold (around 2.2 mM each); above this limit, spontaneous precipitation occurs independently of cellular activity. A preliminary test on a cell-free plate is therefore recommended to distinguish active, cell-driven mineralization from passive calcium/phosphate precipitation caused by supersaturation.

In 2D cultures, VIC and VEC mineralization assays are typically conducted in 48-well plates. The OM is selected based on the study's objectives. Mineralization duration depends on both OM composition and donor variability, as cells from different donors show heterogeneous sensitivity to mineralization.

#### 3.2.3 Mimicking VECs phenotypic transition

TGF-β1 is the most commonly used cytokine to induce EndMT in VECs *in vitro*. Typical concentrations range from 1 to 5 ng/ml for long-term exposure (1–14 days) to 100 ng/ml for short-term treatments (15, 101–103). TGF-β1 rapidly (2–5 days) and persistently (up to 14 days) increases α-SMA while progressively downregulating endothelial markers like VE-Cadherin. After 14 days, it also enhances ALP activity and upregulates osteogenic markers such as OPN, OCN, and RUNX2 (102). Similarly, IL-6 and TNF-α (100 ng/ml) promote EndMT through an Akt/NF-κB-dependent pathway (15). Interestingly, culturing VECs in OM induces a myofibroblastic phenotype within 7 days, followed by a gradual osteogenic transition by day 14 (102).

### 3.3 Reproducing comorbidities *in vitro*

*In vitro* models can integrate patient comorbidities to improve translational relevance. For example, VICs and VECs can be compared based on donor sex (79, 104), age (78), or valve morphology (bicuspid vs. tricuspid) (89, 105) to investigate how these variables influence valvular cell physiology and drug response. In addition, the effects of circulating factors can be evaluated by exposing healthy cells to patient-derived serum (106). Indeed, we observed that serum from AS patients increases VIC calcification compared to non-stenotic controls, thereby providing a relevant system to evaluate the protective effects of new drug candidates (100). This strategy is particularly valuable for modeling comorbidities like CKD or diabetes, where circulating toxic compounds present in the serum—such as uremic toxins, phosphate, glucose, or advanced glycation end-products—contribute to valvular degeneration. In our hands, adding 1%–2% human serum to OM supports mineralization, while exceeding 10% compromises cell viability. The specific effects of pathological factors—including inflammatory cytokines (100), advanced glycation end-products (107), glucose (108), uremic toxins (109), hypoxia (110), neutrophil extracellular traps (NETs) (111) and oxidized lipoproteins (112)—can be evaluated by exposing VICs or VECs to each factor, with or without OM.

### 3.4 Mimicking cellular interactions

#### 3.4.1 VICs-VECs interactions

VICs-VECs communication is essential for maintaining leaflet homeostasis. Using a transwell co-culture system (without direct contact), Hjortnaes et al. showed that VICs attenuate TGF-β1-induced EndMT in VECs, evidenced by reduced α-SMA expression. Similar results were observed when VECs were exposed to VIC-conditioned medium (CM) (102). When co-cultured in Transwell systems, VICs also inhibited the osteogenic transition of VECs under OM, suggesting a protective role in VEC physiology. Conversely, VECs not only failed to inhibit, but actually promoted OM-induced osteogenic differentiation and calcification in VICs (102). These findings highlight the value of *in vitro* systems that incorporate intercellular communication for advancing our understanding of AS pathophysiology.

#### 3.4.2 VICs-macrophages interactions

Co-culture systems and CM have also helped to elucidate VIC-macrophage communication. In 2017, Li et al. showed that CM from pro-inflammatory macrophages enhances VIC osteogenic transition. This effect was blocked when antibodies targeting TNF-α or IL-6 were added to the system; demonstrating the role of inflammation in this process (113). In 2020, Raddatz et al. demonstrated that direct co-culture with macrophages elevated RUNX2 expression in VICs compared to transwell co-cultures, highlighting the role of physical contact between cells in AS pathogenesis (114). More recently, Xia et al. found that EVs from pro-inflammatory macrophages, once internalized by VICs promote osteogenic transition and mineralization more strongly than EVs from control macrophages (115). These models collectively demonstrate how macrophages influence VIC phenotype via cytokines, contact, and EVs and how *in vitro* systems can dissect these complex interactions.

### 3.5 Three-dimensional (3D) models

Mechanical cues from the ECM (including its composition, stiffness and stretch) influence VIC phenotype and disease progression (116). VICs sense stiffness via focal adhesions, adjusting integrin expression and cytoskeletal organization to maintain force balance (117, 118). These mechanosensing processes also modulate intracellular signaling, making VICs highly responsive to their mechanical environment (119). While widely used, 2D cultures fail to replicate the complexity of the native tissue microenvironment, particularly mechanotransduction and cell-ECM interactions, which are critical for VIC regulation. In addition, culturing VICs on polystyrene triggers spontaneous activation and pathological differentiation, limiting the physiological relevance of 2D models in CAVD research. To address these shortcomings, 3D culture systems-with or without scaffolds-have been developed (Table 2).

**Table 2:** Overview of key 3D cellular models currently available to study CAVD.

3D model type		Model name	Principle	Advantages	Limitations	Key findings
Scaffold-free models		Spheroids	VICs self-assemble into three-dimensional aggregates without a pre-formed scaffold when seeded in non-adherent substrates	- High reproducibility - Recapitulates VIC aggregation, myofibroblastic differentiation, apoptosis, and calcium accumulation (120, 121) - Allows study of dystrophic calcification (38,201,249/28,128,382) - Models healthy AV when cultured with ascorbic acid, which maintains VIC phenotype (122)	- Lacks full native 3D tissue architecture and hemodynamic context (120–122) - Substrate rigidity influences VIC aggregation, differentiation, and spheroid calcification (121)	In models of dystrophic calcification, TGF-β is not required for calcification itself but primarily facilitates cell aggregation (121)
		Magnetic levivation	Magnetic nanoparticles are internalized by cells, which are then levitated at the air–liquid interface using a magnetic field, rapidly forming 3D aggregates (∼4 h)	- Suitable for monocultures of VICs or VECs (123) - Sequential assembly of VEC and VIC layers using a magnetic rod (VEC first, then VIC) enables co-cultures mimicking the native AV structure (123) - Preserves VIC and VEC phenotypes (123) - Suitable for studying EndMT (123)	- Lower ECM gene expression vs. 2D (123) - Lack of scaffold prevents replication of native AV architecture (123)	Not yet applied for mechanistic discoveries
Hydrogels	Naturally derived	HAMA hydrogel	Methacrylation (MA) of hyaluronic acid (HA) allows UV-induced crosslinking into a stable 3D HAMA scaffold	- Supports VIC phenotype maintenance (124) - Slow degradation, suitable for long-term experiments (124)	Not reported	Not yet applied for mechanistic discoveries
		GelMA hydrogel	Methacrylation (MA) of gelatin (Gel) allows UV-induced crosslinking into a stable 3D GelMA scaffold	Supports VIC phenotype maintenance (125) - Suitable to study VIC myofibroblastic transition in response to TGF-β1 (125) - Supports VIC and VEC co-culture in a 3D environment (126)	- Rapid degradation - Prone to contraction by aVICs	High glucose upregulates osteogenic transition of both VICs and VECs (126) VECs exert a protective role against VIC osteogenic transformation (126)
		Hybrid HAMA–GelMA hydrogel	Scaffold composed of hybrid HAMA–GelMA hydrogels	- Maintains quiescent VIC phenotype (127) - Suitable to study VIC myofibroblastic transition in response to TGF-β1 (127) - Suitable to study VIC osteogenic transition (127) - Slower degradation than GelMA alone	Not reported	The myofibroblast state precedes osteogenesis in CAVD (128)
		Collagen-based hydrogel	pH neutralization and thermal gelation of collagen I induce fibrillogenesis and formation of a 3D hydrogel network	- Supports VIC and VEC co-culture in a 3D environment (129) - Supports tri-culture with VIC, VEC and macrophages (130) - Collagen matrix can be enriched with other ECM components to better mimic native AV (129) - Supports equibiaxial constraint to study matrix compaction by VICs/VECs (131)	Not reported	- Chondroitin sulfate enhances VIC myofibroblast transition, VEC EndMT, and calcific nodule formation (129) - HA promotes VEC invasion but does no trigger EndMT or calcification (129) - VICs compact the collagen matrix, whereas VECs do not (131) - VECs prevent VIC activation and matrix compaction (131) - In collagen matrices, VIC–VEC co-culture promotes calcification (131) - VECs migrate to calcified nodules and undergo EndMT, contributing to calcific remodelling (131) - Monocytes and M1-like macrophages enhanced ROS-induced calcification of VICs/VECs co-cultured in 3D (130) - M1-like macrophages counteract ROS-induced suppression of VIC-mediated matrix remodelling (130)
		Bioprinted hydrogels	VECs and/or VICs are encapsulated in alginate–gelatin hydrogels and printed layer-by-layer via extrusion-based 3D bioprinting (parallel lines or honeycomb) to recreate the native AV architecture; constructs are crosslinked with CaCl_2_ and cultured up to 21 days	- Multicellular, anatomically relevant 3D model - Customizable architecture - Supports long-term cell viability even in thick constructs (132, 133) - Bioprinted models recapitulate 94% of the CAVD proteomic signature, versus 70% in traditional 2D cultures (134)	Requires specialized 3D bioprinter	Revealed novel proteins in AV calcification (134)
	Synthetics	PEG hydrogel	PEG hydrogels are formed through light-induced radical polymerization, enabling cytocompatible crosslinking and facile functionalization for cell encapsulation	- Supports cell–matrix interactions by incorporating ECM-derived peptides and MMP-degradable sequences, promoting VIC spreading, proliferation, and migration (135, 136) - Suitable to study VIC myofibroblastic transition in response to TGF-β1 (136) - Suitable to create functionalized PEG-constructs with embedded VICs and surface-seeded VECs, supporting layer-specific ECM production (137) - Crosslinking density modulates VIC myofibroblast differentiation (138, 139)	Not reported	- Macrophages can drive VIC myofibroblast-to-osteogenic transition (140) - VICs from non-calcified tricuspid AV regions exhibit higher basal tonus than VICs from corresponding calcified regions (105) - In BAVs, VICs from the raphe region show higher basal tonus than non-raphe regions (105) - VICs from female patients exhibit higher basal tonus than those from males (105) - VECs protect VICs from myofibroblastic differentiation (139) and osteogenic transformation (137)
		Laminar paper-based hydrogel culture system	Hybrid hydrogels combining natural collagen I or HA with PEG for tunable bioactivity and mechanics are polymerized in wax-printed wells on filter paper sheets, then stacked into multilayer constructs mimicking native valve leaflet architecture (141)	Allows recreating “healthy” (HA-rich) versus “pathological” (collagen-rich) ECM environments by varying the ratio and spatial distribution of collagen and hyaluronan (141)	Not reported	Collagen-rich, pathological constructs induced myofibroblastic activation and osteogenic differentiation, whereas HA-rich, healthy-like layers preserved cell viability and suppressed calcific markers (141)
Other types of scaffolds		Electrospun scaffolds	- Electrospinning fibers onto a cold (−30 °C) drum creates ice-templated scaffolds; lyophilization removes ice crystals, leaving a porous 3D fiber matrix that mimics native tissue mechanics and structure.	- Produces fibers from nano- to micrometer scale, enabling layered scaffolds with varied pore sizes and densities that replicate fibrosa, spongiosa, and ventricularis structures (142) - Biofunctionalizable with ECM proteins (142) - Supports mono- and co-culture setups (142) - Suitable for studying VIC activation and calcification (142) - Compatible with perfusion and dynamic culture systems (142)	Not reported	Not yet applied for mechanistic discoveries

This table summarizes the main methods used to investigate VIC and VEC pathophysiology in three dimensions, detailing their principles, advantages, limitations, and key findings obtained so far with these models.

#### 3.5.1 Scaffold-free 3D models

##### 3.5.1.1 Spheroids

Spheroids are three-dimensional, self-assembled cell aggregates that mimic tissue-like interactions. In recent years, these systems have been used to study how VIC myofibroblastic differentiation and apoptosis drive dystrophic calcification. In 2017, Roosen et al. created spheroid structures by seeding porcine VICs into non-adherent agarose microwells, prepared using a 3% agarose solution cast into biocompatible silicone molds, and cultured them in standard medium (122). With this initial protocol, aggregates rapidly degenerated, showing early signs of cell death and mineralization. To address this, the authors supplemented the culture medium with 250 μM ascorbic acid, an essential nutrient and antioxidant, which enabled the formation of viable, high-quality aggregates with no signs of degeneration or calcification. ECM analysis revealed significant increases in GAG, elastin, reticular fibers, and collagen I over the culture period. Aggregates also showed enhanced mRNA expression of Col I/III/V, elastin, hyaluronan, biglycan, decorin, versican, MMP-1/2/3/9, and TIMP-2 compared to monolayer cultures. VICs in aggregates displayed lower α-SMA expression, while osteogenic and chondrogenic markers (OCN, Egr-1, Sox-9, Runx2) remained unchanged, demonstrating that this 3D approach overcomes VIC activation in 2D and promotes a quiescent VIC state. In 2024, Coutts et al. used this model to study the process of dystrophic calcification (120). They applied the protocol developed by Roosen et al. without supplementing the medium with ascorbic acid and observed the formation of calcium nodules within the spheroids after only a few days. Inhibition of apoptosis using Z-VAD markedly reduced calcification, confirming that the process was dystrophic rather than osteogenic. In 2017, Cirka et al. used a different technique to form spheroids, seeding VICs onto collagen-coated micro-contact printed areas on polyacrylamide gels, where the cells self-assembled into aggregates with diameters ranging from 50 to 400 μm (121). These aggregates exhibited myofibroblastic markers, apoptosis, and calcium accumulation. Their exposure to the pan-caspase inhibitor Z-VAD-FMK reduced calcification by approximately 75%, confirming the dystrophic nature of the process. Using this system, the authors showed that TGF-β treatment was not required for calcification itself but primarily facilitated cell aggregation. Interestingly, the authors observed that calcification occurred when aggregates were formed on polyacrylamide gels with stiffness ranging from 9.6 to 76.8 kPa, highlighting the importance of substrate rigidity as a tunable support influencing VIC aggregation, myofibroblastic differentiation, and calcification in this model.

##### 3.5.1.2 Magnetic levitation

In 2014, Tseng et al. used magnetic levitation (Bio-Assembler Kit, Nano3D Biosciences) on VICs, VECs, and their co-cultures to form multilayered cellular constructs, introducing a novel 3D model for AV research (123). Confluent monolayers were incubated with magnetic nanoparticles, detached, and then seeded into ultra-low attachment plates. A magnetic driver positioned above the plate levitated cells to the air-liquid interface, forming 3D cultures within 4 h. VIC and VEC layers were sequentially assembled using a magnetic rod–the VEC layer first, followed by the VIC layer–to create a co-culture mimicking AV structure. The construct was stabilized in VEC medium, then transferred and re-levitated in 24-well plates. Immunohistochemistry and qRT-PCR confirmed preservation of cell phenotype, with CD31 (VEC) and αSMA (VIC) expression. ECM proteins such as collagen type I, laminin, and FN were detected, though gene expression was lower than in 2D cultures. Reduced expression of collagen type I, lysyl oxidase, and αSMA in co-cultures suggested VICs quiescence. Co-localization of CD31 and α-SMA hinted at potential EndMT, suggesting that this model may provide a robust platform for studying AV biology and disease mechanisms. However, the absence of a defined scaffold limits its ability to replicate the AV's complex structure. Hydrogels partly address this limitation by offering a tunable 3D matrix that supports cell interactions and ECM biofunctionalization.

#### 3.5.2 Hydrogels

Over the last decade, photopolymerizable hydrogels have gained attention in the field of 3D culture due to their elastic structure, which allows efficient VIC encapsulation, and their composition, which provides molecular cues essential for cell behavior and differentiation. Both natural and synthetic hydrogels have been explored as cell carriers, considering that an effective scaffold for valvular cells must support adhesion, proliferation, ECM production, and allow investigation of VIC/VEC phenotypic transitions.

##### 3.5.2.1 Naturally derived hydrogels

Naturally derived hydrogels based on gelatin (Gel) and hyaluronic acid (HA), both key factors for VIC adhesion and proliferation (124), have been widely used for AV tissue engineering. Gelatin is a denatured form of collagen, and HA is the predominant GAG in AV ECM, known for its elasticity and specific interactions with FN. In their native form, Gel and HA are soluble and non-crosslinked, limiting their application as scaffolds. To overcome this, researchers methacrylated (MA) them into GelMA (143) and HAMA (124), allowing UV-induced crosslinking into stable 3D structures that preserve their bioactivity. VICs seeded on HAMA spread, proliferated, and formed a confluent monolayer within four days. HAMA preserved HA's ability to bind FN, enabling the design of gels containing both HA and FN, which enhanced ECM production and supported VIC phenotype maintenance (124). Its slow degradation rate (144) makes it suitable for long-term experiments, including those focused on calcifications. Similarly, VICs seeded in GelMA regained their native morphology within 2 weeks, a process accelerated by TGF-β1 (125), which also promotes aVICs formation and collagen-1 synthesis. However, GelMA alone degrades rapidly, limiting its use in extended studies. To address this, a hybrid HAMA-GelMA hydrogel was developed, improving stability and better mimicking the native AV ECM (127). In this system, VICs remain quiescent unless stimulated with TGF-β1 and differentiated into first aVICs and then obVICs when exposed to an OM (128). Silencing α-SMA reduced both osteogenic differentiation and calcification, suggesting that the myofibroblast state precedes osteogenesis in CAVD.

##### 3.5.2.2 Synthetic hydrogels

Natural 3D matrices closely mimic physiological environments, supporting VIC viability, remodeling, and acting as reservoirs for bioactive molecules. However, their complexity may obscure specific cell-matrix interactions.

To overcome this, Benton et al. introduced in 2009 synthetic hydrogels based on polyethylene glycol (PEG), a synthetic, non-toxic, non-immunogenic, polymer approved by the FDA, which can be easily functionalized for cytocompatible encapsulation via light irradiation. PEG hydrogels are highly hydrated and mimic the mechanical properties of soft tissues. To enhance cell-matrix interactions, they incorporated RGD peptide (derived from FN) and crosslinked the PEG with a MMP-degradable sequence (GPQGIWGQ), enabling cell-driven remodeling. This system supported integrin αvβ3 binding, as well as cell spreading, proliferation, migration, and TGF-β1-induced myofibroblast differentiation (136). In 2016, Gould et al. encapsulated VICs in PEG hydrogels functionalized with ligands derived from FN (RGDS), elastin (VGVAPG), or collagen-1 (P15). VICs cultured in FN-functionalized hydrogels showed higher MMP activity at day 2 and exhibited elongation by day 14 compared to those in elastin or collagen-1 gels. The highest proportion of αSMA + VICs was observed in elastin gels (56%), compared to FN (33%) or collagen (38%); along with higher collagen-X:collagen-I ratio, a marker associated with stenotic valves (135). In 2020, Grim et al. demonstrated using PEG hydrogels that CM from pro-inflammatory macrophages promotes the osteogenic transition of activated VICs cultured in 3D (140). They reported that this effect was mediated by the secretion of TNF-α, IL-1β, and IL-6, suggesting that macrophages may drive a myofibroblast-to-osteogenic shift in VICs, thereby linking fibrosis to calcification in AS. In 2023, Tuscher et al. used PEG hydrogels to characterize the basal contractile behavior of VICs from tricuspid AVs. To do so, they tracked VIC-induced gel displacements and shape changes after treatment with Cytochalasin D, an actin polymerization inhibitor that depolymerizes VIC stress fibers. They demonstrated that VICs from the non-calcified region of tricuspid AV were significantly more activated than those from the corresponding calcified regions. Moreover, when studying VICs from bicuspid AVs, they showed that cells from the raphe region were more activated than those from non-raphe regions. Changes in VIC morphology following Cytochalasin D treatment indicated that cells from tricuspid valves and BAVs possess distinct cytoskeletal structures, providing new insights into the cellular mechanics underlying CAVD progression in BAV patients (105). This model also revealed that VICs from female patients exhibited significantly higher basal tonus levels than those from male patients.

##### 3.5.2.3 Co-cultures in hydrogels

Over the years, hydrogel-based co-culture models have been developed to better mimic the native AV environment. In 2015, Puperi et al. created an endothelialized AV model using PEG-RGDS and PEG-PQ (MMP-2/MMP-9 degradable) hydrogels seeded with VICs. The surface was functionalized with RKR (a laminin-derived syndecan-binding peptide) to support VEC adhesion. In this model, VECs formed a CD31+ monolayer with minimal αSMA activation within 7 days. VICs in 3D PEG-PQ showed reduced αSMA expression compared to 2D culture, an effect amplified in co-culture. By day 28, VECs secreted basement membrane components (laminin, perlecan, and collagen type IV), while VICs produced collagen and FN, suggesting layer-specific ECM production (137). In 2022 Bramsen et al. co-cultured VICs and VECs in collagen I hydrogels enriched with chondroitin sulfate (CS) and HA, two GAGs typical of the spongiosa (129). CS enhanced VIC myofibroblast transition, VEC EndMT, and calcific nodule formation, while HA promoted VEC invasion without triggering EndMT or calcification. This observation underscores the influence of ECM composition on valvular cell fate, further supporting the relevance of 3D models for preclinical studies.

In 2014, Gould et al. showed that increasing PEG hydrogel stiffness by raising crosslinking density promoted VIC myofibroblast differentiation, an effect abolished by VEC co-culture due to paracrine NO signaling (139). In contrast, Mabry et al. found that increasing hydrogel stiffness suppressed VIC activation (138). This inverse relationship suggests that while matrix stiffness is a key regulator of VIC phenotype, its effects are context-dependent and strongly influenced by the method used to modulate stiffness. Unlike Gould, Mabry et al. increased stiffness using a secondary non-degradable network in a thiol-ene hydrogel system, which enabled to decouple stiffness from network density. This approach provided independent control over mechanical properties without altering mesh size or cell morphology—two factors often affected when stiffness is tuned by increasing crosslinking. By isolating mechanical cues from structural variables, their system allowed a more precise investigation of how stiffness influences VIC behaviour and differentiation. In 2021, Gee et al. studied porcine VEC and VIC responses under tension using equibiaxially constrained collagen hydrogels. VICs compacted the matrix, while VECs did not. Co-culture reduced VIC activation and matrix compaction, though OM reversed these effects. OM also induced calcified nodule formation, further enhanced by VEC co-culture. Inhibiting canonical NFκB reduced calcification but not fibrosis, indicating its specific role in osteogenic remodeling. Immunofluorescence revealed that VECs clustered on calcified nodules and expressed EndMT markers, suggesting that EndMT contributes to calcific remodeling (131).

Finally, GelMA constructs with embedded VICs and surface-seeded VECs were recently used to model CAVD under diabetic conditions (126). The use of these models allowed researchers to demonstrate that high glucose upregulates osteogenic markers through the TGF-β and BMP-2 pathways in both cell types, leading to increased calcium deposition. Exposure to OM further enhanced calcification in VIC-only constructs compared to VIC–VEC constructs, confirming the protective role of VECs against VIC osteogenic transformation.

Going further in complexity, Salemizadehparizi et al. established in 2025 a tri-culture model of the fibrosa, in which THP-1 monocytes or M1-like macrophages were seeded atop collagen-I constructs containing embedded VICs and surface-seeded VECs (130). The VIC/VEC model was pretreated with H_2_O_2_ for 7 days before the addition of monocytes or M1-like macrophages, and was then maintained for 14 days, allowing assessment of the combined effects of ROS and immune cells while avoiding H_2_O_2_ toxicity to monocytes/macrophages. Using this system, the authors showed that monocytes and M1-like macrophages enhanced ROS-induced calcification compared to co-cultures without immune cells. Monocyte-tri-cultures formed smaller, more circular nodules, whereas M1-tri-cultures formed nodules of intermediate size and morphology, indicating that inflammatory macrophages modulate calcified deposit structure. Moreover, while H_2_O_2_ inhibited hydrogel contraction in VIC-only, co-culture, and monocyte-tri-culture models, contraction persisted in M1-tri-cultures, suggesting that inflammatory macrophages can counteract ROS-induced suppression of VIC-mediated matrix remodeling.

Data obtained with these co-culture models underscore how interactions between cells and the ECM shape VEC–VIC communication and contribute to CAVD development.

##### 3.5.2.4 Toward tri-layered constructs

The tunability of 3D hydrogels offers precise control over ligand and peptide incorporation, cell–material interactions, stiffness and matrix degradability. However, most models lack the AV's trilayered structure and dynamic complexity. In this context, Monroe et al. developed in 2019 a 3D laminar paper-based culture system to dissect how ECM composition directs VIC behavior in CAVD (141). Porcine VICs were encapsulated in hydrogels containing either collagen I or hyaluronan, with both matrices functionalized with PEG-linked peptides and engineered to have equivalent shear mechanics to isolate biochemical effects. The gels were polymerized in wax-printed wells on filter paper sheets, which were then stacked into multilayer constructs mimicking native leaflet architecture. By varying the ratio and spatial distribution of collagen and hyaluronan, the authors recreated “healthy” (HA-rich) vs. “pathological” (collagen-rich) ECM environments. VICs remained viable and proliferative under both conditions; however, collagen-rich, pathological constructs induced higher α-SMA and RunX2 expression, indicating myofibroblastic activation and osteogenic differentiation. In contrast, HA-rich, healthy-like layers preserved cell viability and suppressed the expression of calcific markers, highlighting the critical influence of ECM composition on VIC phenotype. This system demonstrates the power of 3D biomimetic platforms to model both normal and disease-like valve microenvironments.

More recently, Immohr et al. introduced a 3D bioprinting strategy to engineer multicellular, anatomically accurate AV constructs, providing a promising platform for modeling CAVD and drug screening (132, 133). To achieve this, they encapsulated porcine VICs and/or VECs within alginate- and gelatin-based hydrogels. These cell-laden hydrogels were then deposited layer-by-layer using extrusion-based 3D bioprinting according to defined patterns (parallel lines or honeycomb structures) to recreate the native three-dimensional architecture of the AV. After printing, the constructs were stabilized by crosslinking in a calcium chloride solution and subsequently cultured for up to 21 days under standard conditions to maintain cell viability and enable cellular interactions within this multicellular 3D model. Good long-term cell viability was confirmed even in thick, multilayered multicellular constructs, providing proof of principle that 3D bioprinting of VEC- and VIC-based hydrogels is a feasible approach to design constructs that mimic the native AV. In 2024, Clift et al. used this type of 3D bioprinting approach to encapsulate human VICs within GelMA/HAMA-based hydrogels and compare their cellular proteome and vesiculome with those of human CAVD tissues (134). Liquid chromatography–tandem mass spectrometry analyses showed that the bioprinted model recapitulated 94% of the CAVD proteomic signature, vs. 70% in traditional 2D cultures. Integration of cellular and vesicular datasets revealed both known and previously unrecognized proteins linked to AV calcification. This study confirms that 3D bioprinted cellular models more faithfully reproduce human disease biology than 2D systems, offering a robust platform for high-throughput multiomics studies and drug discovery.

#### 3.5.3 Electospun scaffolds

In addition to hydrogels, cryogenic electrospinning has recently emerged as a powerful method for creating 3D tissue models. This technique involves electrospinning fibers onto a cold drum (−30 °C), where water vapor forms ice crystals that serve as a temporary void template. Lyophilisation removes the ice, leaving a loosely packed fiber structure. The resulting scaffold mimics native tissue mechanics and cell distribution, offering a more physiologically relevant environment for studying processes like valve calcification.

In 2022, Stadelmann et al. developed a bi-layered cryogenic electrospun scaffold using polylactic acid, a biodegradable, biocompatible polymer commonly used in tissue engineering (142). The technique produced fibers ranging from nanometers to micrometers, forming two layers: a bottom infiltration layer (IL) with large pores, mimicking the spongiosa and promoting VIC infiltration, and the top non-infiltration layer (n-IL) with dense nanofibers, replicating the fibrosa and supporting VEC adhesion. Both layers were biofunctionalized with ECM proteins (FN, laminin, collagen type I, and fibrin) to support cell-specific needs. In co-culture, VICs were seeded on the IL and VECs on the n-IL, with cultures maintained for 3–6 days and medium changes every 2–3 days. This scaffold showed excellent stability for up to 4 weeks. VICs adhered, maintained viable, migrated into the IL, and displayed fibroblast-like morphology. VECs retained a cobblestone morphology and stayed on the surface of the n-IL. By day 6, both cell types formed confluent layers and expressed appropriate adhesion and cell-contact markers, with only minor variations compared to monocultures. Under osteogenic stimulation, VICs formed calcific nodules and upregulated markers like RUNX2, highlighting the model's suitability for studying VIC osteogenic transition and calcification. This bilayer scaffold, which is compatible with perfused and dynamic systems, provides a promising platform for studying both early and long-term mechanisms of the disease.

## 4 *Ex vivo* modelling of CAVD

While *in vitro* models have advanced our understanding of CAVD, they fall short of replicating the multicellular and matrix complexity of the AV. *Ex vivo* AV cultures provide a valuable intermediate, preserving native architecture while eliminating systemic variables inherent to *in vivo* models.

### 4.1 Porcine models

Most *ex vivo* models use porcine AV due to their structural and compositional similarities to human valves, their healthy state, and their larger size, which facilitates gene and protein expression analysis. Porcine AV mineralization can be induced *ex vivo* by culturing AVs for at least 8 days in medium supplemented with 3.8 mM Pi. The addition of inorganic pyrophosphatase, which degrades PPi, further enhances mineralization (145). These culture conditions preserve the structural integrity of the valves and maintain cell viability, supporting the model's relevance. This model was used in 2021 to evaluate whether etidronate, a PPi analogue, could prevent mineralization. The study demonstrated that etidronate effectively inhibited mineralization, confirming the model's suitability for pharmacological screening (146). In 2014, Rodriguez et al. used porcine AV cultured *ex vivo* to evaluate the role played by the ECM in VIC physiology. To do so, they enzymatically degraded leaflet collagen and studied the impact on VIC phenotype. They observed that ECM disruption triggers VIC proliferation, apoptosis, and the expression of markers like α-SMA, ALP, and OCN, all associated with increased calcification (147).

A key limitation of *ex vivo* models is maintaining tissue viability for extended periods, which is often challenged by limited nutrient diffusion, ECM degradation, and leaflet contraction caused by myofibroblast activation. This is why, in 2020, Zabirnyk et al. chose to optimize the model by culturing porcine leaflets in an anti-myofibroblastic medium. They demonstrated that this medium preserved tissue structure and prevented the typical ball-like contraction observed with standard media (148), making it suitable to study β-GP-induced mineralization. Using this model, they showed that SNF472, an agent inhibiting the formation and growth of hydroxyapatite crystals, effectively prevents AV mineralization.

### 4.2 Other models (human, ovine and murine)

While porcine AVs remain the standard for *ex vivo* culture, human and ovine valves can also be used. As with porcine models, their structure is preserved in antimyofibroblastic media, and mineralization can be induced with OM and reversed pharmacologically, highlighting their potential for drug testing (149, 150). To our knowledge, no standardized model currently exists for studying the remodeling of rodent AV cultured *ex vivo* under static conditions. This may be related to the small size of murine AV, which complicates dissection, culture, and downstream gene and protein analyses, thereby limiting their utility for mechanistic studies. Nevertheless, developing such models would be useful for mechanistic investigations and the screening of new therapeutic molecules. The study by Jenke et al., published in 2020, provides an elegant example of how *ex vivo* models can advance our understanding of CAVD pathophysiology (151). In this study, the authors exposed ovine AV leaflets, mounted and cultured under passive tension on synthetic rubber rings, to TGF-β1, in the presence or absence of OM. Using this protocol, they showed that in a 3D leaflet model, TGF-β1 completely suppresses OM-induced mineralization while promoting fibrosis. This effect was associated with downregulation of osteocalcin and ALP expression and upregulation of α-SMA and collagen I expression. By contrast, they reported that in ovine VICs cultured in 2D, TGF-β1 promoted calcification, demonstrating that cellular responses differ profoundly between 2D cultures and native-like 3D tissue environment.

## 5 Replicating mechanical and hemodynamic conditions

The AV is a dynamic structure that opens and closes each cardiac cycle, exposed to a complex mechanical environment involving cyclic stretch, bending, pressure, and shear stress. Alterations in these forces contribute to the onset and progression of AV dysfunction and remodeling. While static *in vitro* and *ex vivo* models have advanced our understanding of CAVD, they fail to replicate physiological mechanical and hemodynamic conditions. To address this, researchers have developed preclinical systems allowing investigation of mechanical and hemodynamic influences on valvular cells and tissues (Table 3).

**Table 3:** Experimental platforms to reproduce aortic valve mechanical and flow conditions *in vitro* and *ex vivo*.

Sample type	Model name	Parameter reproduced	Principle	Advantages	Limitations	Key findings
VICS/VECS cultures (2D)	Flow-based culture system (IBIDI, Bioflux)	Shear stress	Simulates shear stress in VICs or VECs seeded in microchannel plates, where fluid is pumped at controlled flow rates to mimic physiological conditions	- Precise control of flow regimes - Usually contains real-time imaging tools for dynamic monitoring of cell behaviour - Allows mechanistic studies on isolated cells	- 2D culture does not fully reproduce 3D tissue architecture - Lacks other mechanical forces such as pressure and stretch	- VECs align perpendicularly to the flow (152) - VICs calcification increases when exposed to CM from VECs subjected to increased shear stress (153)
	Flexercell® cyclic strain system	Equibiaxial cyclic strain	VICs or VECs are cultured on flexible collagen-coated membranes (BioFlex® plates) cyclically deformed by vacuum pressure, applying controlled cyclic strain	- Precise control of strain magnitude and frequency - Allows mechanistic studies on isolated cells	- 2D culture does not fully reproduce 3D tissue architecture - Lacks shear stress	Physiological strain (10%–15%) prevents the inflammatory activation of VICs (154) and VECS (155).
	Cell culture on collagen-coated polyacrylamide gels of defined stiffness	Substrate stiffness	VICs, pre-activated on stiff plastic, are cultured on collagen-coated polyacrylamide gels of defined stiffness	Substrate stiffness tunable from soft (∼150 Pa) to pathologically stiff (∼150 kPa)	- 2D culture does not fully reproduce 3D tissue architecture - Lacks shear stress	- High stiffness triggers VIC myofibroblastic activation; low stiffness reverses it (156) - Stiffness is stronger than TGF-β1 for myofibroblastic activation (156)
VICs/VECS in hydrogels (3D)	Parallel plate flow chamber	Shear stress	Simulates physiological laminar shear stress by perfusing fluid over the sample placed in a flow channel between parallel plates, allowing controlled application of wall shear stress comparable to native valve conditions.	Allows evaluation of the impact of shear stress on cells cultured in 3D	Lacks other mechanical forces such as pressure and stretch	- VECs align perpendicularly to the flow (157) - Shear stress activates VICs myofibroblastic differentiation (157) - VECs in dynamic co-culture maintain VIC quiescence and ECM homeostasis (157)
	3D mechanically constrained hydrogel platform	Tissue compaction, stress and stiffness	VICs encapsulated in collagen hydrogels suspended between PDMS posts	- Enables real-time measurement of tissue compaction, cell-generated tension, stiffness, and local protein expression (light sheet microscopy)	Lacks perfusion	- Osteogenic stimulation induces neo-tissue compaction, formation of dense surface lesions, disrupted homeostatic stress, and enhanced myofibroblastic activity (158) - Growth factors modulate gene expression independently of tissue stress (158)
	Cell-based three-dimensional valve-on-chip microphysiological system	Mechanical strain	VICs were embedded in layered collagen–GAG and collagen hydrogels to mimic spongiosa and fibrosa, stabilized by UV crosslinking. VECs were seeded on the fibrosa surface to form a confluent endothelial layer. The construct was housed in a PDMS chamber on an elastic membrane to allow controlled mechanical strain	- Collagen and GAG content can be adjusted to mimic healthy or diseased valves - Constructs can be exposed to either quiescent or osteogenic media - VOC composition and culture conditions can be tailored to produce healthy constructs (healthy hydrogel + quiescent media) or diseased constructs (diseased hydrogel + pro-osteogenic media). - Constructs are mounted on an uniaxial stretcher to apply healthy of pathological cyclic strain	Lacks perfusion	- Healthy hydrogels maintain VIC quiescence, while diseased hydrogels induced myofibroblast activation (159) - Proteins involved in cell cycle or cholesterol biosynthesis are altered in diseased conditions (159)
AV leaflets (usually porcine leaflets)	Double cone-and-plate system	Shear stress	Rotation of the cones over a flat plate generates controlled shear stress on both leaflet sides, mimicking side-specific hemodynamics	Allows evaluation of the impact of shear stress on native AV leaflet	- Complex setup - Lacks other mechanical forces such as pressure and stretch	- Side-specific effects of shear stress on ECM composition (160) - Sensitivity of AV leaflets to both the intensity and frequency of shear stress (161)
	Cyclic pressure bioreactor	Cyclic pressure (mimicking diastolic loading)	Applies controlled cyclic pressure to valve leaflets *ex vivo* to simulate physiological or hypertensive diastolic pressure conditions.	- Suitable to study pressure-induced biological responses in intact leaflets - Mimics hypertensive stress relevant to AS	Limited to pressure application without shear stress	Hypertensive pressure (120 mmHg) upregulates inflammation-related genes in VICs (162)
	Cyclic stretch bioreactor	Cyclic radial and circumferential stretch	Applies controlled cyclic stretch to AV leaflets suspended between fixed and moving posts, mimicking physiological or hypertensive deformation patterns.	- Suitable to study cyclic stretch in intact leaflets - Enables controlled modulation of stretch magnitude and frequency - Useful for studying mechanotransduction and ECM remodeling	No shear stress or pressure simulation	- Cyclic stretch drives ECM remodeling and VIC phenotypic activation (163) - Synergistic effect of cyclic stretch and TGF-β1 on VIC activation (164) - BMPs are key mediators of stretch-induced leaflet calcification (165)
	Biaxial micromechanical testing system	Equibiaxial mechanical stretch	Leaflets are mounted with springs and tracked markers, then subjected to controlled equibiaxial stretch while recording force and deformation via force transducers and a 2D camera system	Provides precise, real-time characterization of tissue mechanics	Does not replicate shear stress or pressure	- Key role of the endothelium in cusp stiffness (166)
	Microphysiological pulsatile flow platform	Pulsatile flow (dynamic hemodynamics)	Combines pulsatile micropumps with a tissue incubation chamber to maintain dynamic *ex vivo* culture of leaflets over 14–26 days, mimicking physiological pulsatile hemodynamics	- Supports long-term culture with preserved metabolic activity, collagen deposition, and contractility - Applicable to both porcine and human tissues - Suitable to study the mineralisation process (160)	- Complex setup - Limited throughput - May not fully replicate all mechanical forces	Not yet applied for mechanistic discoveries
Native AV	Miniature *ex vivo* culture system for murine AV	Flow under *ex vivo* perfusion conditions	Perfusion of intact mouse hearts for 7 days with or without OM	- Preserves native valve architecture and microenvironment - Suitable to study AV calcification under physiological-like conditions	- Flow direction is reversed compared to physiology (from aorta to left ventricle) so the AV remains closed - Altered shear stress distribution	- VICs show different pro-calcific responses to OM in 2D culture compared to 3D perfused AV culture, highlighting the importance of the native microenvironment in calcification (167, 168) - Treatment with SPV106, a histone acetyltransferase activator, reduced AV osteogenic activation and calcification (169)
	Pulsatile organ culture system for porcine AV	Physiological pulsatile flow and pressure mimicking left heart function	Circulation of fluid during 48 h through the AV via piston pump and latex diaphragm in sterile conditions, combined with compliance tank, mechanical mitral valve, and waveform generator to replicate physiological hemodynamics	- Culture under near-physiological hemodynamic conditions - Preserves ECM, cell phenotype, and endothelial integrity - Reduces apoptosis compared to static culture (170)	Complex setup	Role of mechanical stimulation in maintaining AV integrity (170)
	Pulsatile organ culture system for ovine AV	Physiological pulsatile flow mimicking left heart function	Culture of ovine AVs under controlled pulsatile flow for 7 days to mimic physiological hemodynamics	- Enables study of calcification patterns under osteogenic stimulation (171)	- Calcification location differs from human CAVD (ventricularis vs. aortic side) - Limited VIC phenotypic switching (171)	Not yet applied for mechanistic discoveries

This table summarizes the main in vitro and ex vivo models used to mimic the mechanical and hemodynamic environment of the aortic valve, outlining their principles, advantages, limitations, and key findings obtained so far with these models.

### 5.1 *In vitro* approaches

To simulate hemodynamic forces, especially shear stress, on 2D cultures of VICs and VECs, flow-based culture systems such as Bioflux (Fluxion Biosciences) and IBIDI (IBIDI GmbH), have been developed. These platforms use microchannel plates through which fluid is pumped at controlled flow rates, generating shear stress that mimics physiological conditions. They offer precise control over flow regimes and often integrate real time imaging tools for dynamic monitoring of cell behavior. This enables investigation into how mechanical forces affect cell proliferation, apoptosis, differentiation, and response to pathophysiological stimuli.

Using this type of system, Butcher et al. showed that aortic endothelial cells exposed to a steady shear stress of 20 dynes/cm^2^—approximating average physiological levels (172)—aligned parallel to the direction of flow, while VECs aligned perpendicularly, revealing distinct mechanical phenotypes adapted to their respective function (152). Transcriptomic analyses further confirmed that VECs differ from aortic endothelial cells, notably in their higher proliferative capacity (173). More recently, Hsu et al. used this system to investigate how shear stress (1 dyne/cm^2^—mimicking an atherogenic environment) affects VIC calcification (153). To do so, VECs were exposed for 48 h to static, steady, or oscillatory flow (0.25 or 0.5 OSI), and their CM were then added to VICs cultured in an OM. They found that VIC calcification was significantly increased in the 0.5 OSI group, highlighting the pro-calcific effect of oscillatory shear stress. To increase system complexity, Butcher et al. developed 3D collagen hydrogel-based AV models to study VEC/VIC interactions under flow (157). One model contained only VICs, while the other included a VEC layer on the luminal surface. Both were subjected to 20 dynes/cm^2^ shear stress for 96 h. As in 2D cultures, VECs aligned perpendicularly to flow. Shear stress activated VICs myofibroblastic differentiation. In this dynamic co-culture model, VECs reduced VIC proliferation, preserved GAGs, and enhanced protein synthesis, promoting a quiescent VIC phenotype and maintaining matrix homeostasis.

In 2012, Quinlan et al. developed a high-throughput system using collagen-coated polyacrylamide gels with defined stiffness values ranging from very soft (∼150 Pa) to levels exceeding those of diseased aortic valves (∼150 kPa), to study how substrate stiffness influences VIC behavior in 2D (156). Porcine VICs, pre-activated on stiff plastic, were seeded on these substrates and cultured with or without TGF-β1, and cell spreading, morphology, and αSMA expression were quantified. They found that increasing substrate stiffness led to larger cell spread area, more elongated morphology, and a higher fraction of VICs displaying pronounced αSMA-positive stress fibers, indicative of myofibroblastic activation. By contrast, relatively low stiffness levels were sufficient to “deactivate” VICs. Although TGF-β1 slightly modulated αSMA expression, substrate stiffness was the dominant cue driving myofibroblastic activation. This study highlights the importance of controlling substrate stiffness in *in vitro* models of valve physiology and tissue engineering.

In addition, it is also possible to reproduce *in vitro* the mechanical strains experienced by AV leaflets to study their impact on VICs. Using the Flexercell® system, Smith et al. cultured VICs on flexible collagen-coated membranes (BioFlex® plates) that can be cyclically deformed by vacuum pressure (154). This setup applies controlled, cyclic strain (e.g., 5%–20% at 1 Hz), mimicking physiological or pathological mechanical loading, and allows investigation of downstream effects on gene expression, protein production, and calcification. Using this approach, they showed that applying physiological cyclic strain (15%) to porcine VICs reduced inflammation, as evidenced by decreased expression of MCP-1, VCAM-1, and GM-CSF (154). The system also demonstrated that 10% cyclic strain preserved the integrity of porcine VEC monolayers and limited pro-inflammatory protein expression, whereas both lower (5%) and higher (20%) strain levels were detrimental (155).

In 2024, Shih et al. developed a compact 3D mechanically constrained platform to study how VICs remodel their ECM in response to biochemical and biomechanical cues (158). To do so, they encapsulated VICs in collagen hydrogels suspended between polydimethylsiloxane (PDMS) posts, allowing real-time measurements of tissue compaction, stress (the tension generated by cell contraction and matrix remodelling on the posts), and stiffness, as well as visualization of local protein expression via light sheet microscopy. Osteogenic stimulation induced accelerated neo-tissue compaction, formation of dense surface lesions, and disrupted homeostatic stress levels. Both porcine and human VICs exhibited increased myofibroblastic activity (ACTA2, TGFB1, CNN1) under osteogenic conditions, with protein expression localized in banding patterns at the neo-tissue surface and positively correlated with mechanical stress. The addition of growth factors (EGF and FGF) modulated gene expression independently of tissue stress, demonstrating that differentiation can be biochemically altered without changing mechanical state. This platform, which allows simultaneous analysis of mechanical and molecular drivers of pathological remodeling, offers a high-throughput biologically relevant system to study CAVD mechanisms and potential pharmacological interventions.

In recent years, the field of preclinical modeling has been transformed by the emergence of organ-on-chip technology. An organ-on-chip is a microfluidic device that replicates the structure and function of a human organ by integrating living cells within a controlled, dynamic environment, enabling the study of physiology and disease *in vitro*. To date, few of these microfluidic models have been developed to replicate the AV physiology. In 2024, Tandon et al. developed what currently represents the most advanced cell-based three-dimensional valve-on-chip (VOC) microphysiological system (159). The VOC platform consisted of a rectangular PDMS chamber mounted on an elastic membrane, which allowed the application of controlled mechanical strain. Within this chamber, the valvular construct was formed by sequentially assembling hydrogel layers to reproduce the valve's native architecture. Quiescent pVICs (1.5 × 10^6^ cells/ml) were first embedded in a collagen–GAG hydrogel to form a spongiosa-like layer and allowed to gel overnight. A second collagen-only layer containing quiescent pVICs was then added to mimic the fibrosa and left to set for another night. After polymerization, the bilayer construct was UV-crosslinked for 1 min to stabilize the matrix. pVECs were then seeded on the fibrosa surface (300,000 cells/cm^2^) and cultured for 48 h to form a confluent endothelial monolayer. Healthy and diseased valve compositions were simulated by varying the collagen and GAG content. Healthy constructs contained ∼90% collagen with GAGs restricted to the spongiosa layer, whereas diseased constructs contained 50% collagen and fourfold higher GAG concentrations, reproducing the ECM remodeling characteristic of CAVD. Once assembled, the VOCs were cultured in either quiescent or pro-osteogenic medium (supplemented with β-glycerophosphate, dexamethasone, ascorbic acid, and TGF-β1) to induce calcification. The complete construct was then mounted on a custom uniaxial stretcher applying 10% cyclic strain for 48 h, reproducing physiological mechanical forces and enabling the study of dynamic 3D valve responses under healthy and disease-like conditions. While the healthy hydrogel promoted vimentin expression, maintaining VICs in a quiescent phenotype, the diseased hydrogel induced VIC activation into a myofibroblast-like phenotype, as evidenced by positive αSMA expression. Proteins involved in cellular processes such as cell cycle progression, cholesterol biosynthesis, and protein homeostasis were significantly altered and correlated with metabolic changes in diseased VOCs, suggesting that these constructs may serve as valuable tools to study the early, adaptive stages of disease initiation.

### 5.2 *Ex vivo* approaches on isolated leaflets

#### 5.2.1 Assessing the impact of shear stress

The biological response of AV leaflets to controlled shear stress can be assessed by a cone-and-plate system. This setup, composed of a flat plate and a rotating cone positioned just above it, generates well-defined shear forces by moving fluid between the two surfaces and allows for the application of steady or pulsatile flow while minimizing secondary flow artefacts (174). A more advanced version—the double cone-and-plate system—allows simultaneous exposure of both leaflet surfaces (aortic and ventricular), better replicating *in vivo* side-specific hemodynamics. In 2018, Mongkoldhumrongkul et al. used this system to study the effect of side-specific flow on ECM dynamics in porcine AV leaflets (160). Leaflets were subjected to oscillatory or laminar flow for 48 h. Laminar flow promoted elastin synthesis on both sides, while oscillary flow increased collagen and GAG content specifically on the aortic side, underscoring the importance of side-dependent flow in ECM regulation. In 2013, Sun et al. used a double cone-and-plate system to evaluate the sensitivity of AV leaflets to both the intensity and frequency of shear stress, and their role in initiating CAVD-related remodeling (161). They reported that elevated shear magnitude at normal frequency increased BMP-4 and TGF-β1 expression and triggered ECM degradation in porcine AVs. Abnormal frequency at physiological magnitude also induced matrix remodeling. The most pathological response was observed under sustained supra-physiologic shear, peaking at 48 h and persisting through 72 h.

#### 5.2.2 Assessing the impact of pressure

Hypertension is associated with AS *in vivo* (175). To mimic hypertensive conditions ex-vivo, AVs can be cultured *ex vivo* in specialized pressure chambers (176). In 2011, Warnock et al. cultured porcine AV leaflets for 24 h under cyclic pressures of 80 mmHg (physiological) or 120 mmHg (hypertensive) in a pressure chamber mimicking diastolic loading (162). Transcriptomic analysis revealed 56 inflammation-related genes differentially expressed under hypertensive condition, including TNF-α, IL-1α, IL-1β, and a striking 41-fold upregulation of PTX3. These findings suggest that pressure-induced activation of inflammatory pathways in VICs could represent a potential therapeutic target in hypertensive AS.

#### 5.2.3 Biomechanical testing systems (stretch, strain and stiffness)

Under physiological conditions, AV leaflets experience ∼10% circumferential and up to 30% radial stretch, which can increase under elevated pressure. In 2006, Balachandran et al. used an *ex vivo* bioreactor with two chambers containing fixed and moving posts to apply controlled cyclic stretch via an actuator. Porcine AV leaflets were suspended between the posts and subjected to 15% stretch for 48 h. Compared to fresh or statically cultured leaflets, stretched tissues exhibited increased collagen, reduced GAGs and elevated α-SMA expression, indicating a contractile, fibrotic VIC phenotype. These findings suggest that cyclic stretch drives ECM remodeling and VIC phenotypic activation (163). Using the same setup, Merryman et al. demonstrated a synergistic effect of cyclic stretch and TGF-β1 on VIC activation (164). In 2010, Balachandran et al. showed that the induction of a pathological stretch in porcine leaflets cultured in an OM promoted apoptosis, ALP activity and calcification, along with early upregulation of BMP-2, BMP-4, and Runx2 on the fibrosa surface. In this model, inhibition of BMP signaling dose-dependently reduced calcification and ALP levels, highlighting BMPs as key mediators of stretch-induced AV calcification (165). In 2009, El-Hamamsy et al. used a biaxial micromechanical testing system to investigate how the valve endothelium regulates aortic cusp mechanics. Valve samples were mounted in a Krebs bath at 37 °C with continuous O_2_/CO_2_ gassing to mimic physiological conditions. Stainless steel springs were threaded through each side of the cusp to preserve endothelial integrity, and four markers tracked deformation during equibiaxial stretching. Strain was measured using force transducers and a 2D camera system. Serotonin (5-HT) reduced cusps stiffness by 25%, an effect reversed by endothelial removal or L-NAME (a nitric oxide synthase inhibitor). In contrast, endothelin-1 increased stiffness by 34%, which was blocked by cytochalasin-B (an actin polymerization inhibitor). These findings underscore the endothelium's key role in modulating mechanical properties essential for valve function (166).

#### 5.2.4 Toward organ-on-chips

In 2023, Dittfeld et al. developed a microphysiological platform integrating pulsatile micropumps with a tissue incubation chamber to enable long-term *ex vivo* culture of porcine and human AV tissues. When cultured in this device for 14–26 days under dynamic pulsatile conditions, the tissue displayed increased metabolic activity, collagen deposition, and contractility, which are characteristic of early stages of CAVD. GAGs, endothelial and smooth muscle markers, and calcium deposition remained stable (160), supporting the relevance of this platform for preclinical studies.

### 5.3 Reproducing hemodynamics in native AV

While earlier models focused on isolated leaflets, recent advances have enabled *ex vivo* culture of whole native murine, porcine, or ovine AVs under flow conditions mimicking pathophysiological states. In 2021, Kruithof et al. established an *ex vivo* calcification model for intact wild-type murine AV using a miniature tissue culture system. To do so, they perfused mouse hearts for 7 days, with or without OM. In this model, calcification occurred exclusively when the AV leaflets were cultured in an OM supplemented with Pi, whereas treatment with a cocktail composed of β-GP, ascorbic acid, and dexamethasone did not induce calcification. By contrast, they observed that murine VICs cultured *in vitro* exhibited calcification under both Pi and the β-GP–ascorbic acid–dexamethasone cocktail. This study revealed that significant disparities exist between *in vitro* and *ex vivo* responses of VICs, highlighting the added value of investigating CAVD in cells embedded within their native microenvironment (167, 168). However, in this setup, flow was reversed compared to physiological conditions (i.e., directed from the aorta to the left ventricle), keeping the valve closed and continuously exposing the aortic side to hemodynamic stress, which represents a limitation. In 2025, Garoffolo et al. used this model to show that SPV106, a histone acetyltransferase activator, significantly reduced AV calcification and osteogenic marker expression (ALP, RUNX1/2/3), demonstrating the model's utility for preclinical evaluation of anti-calcific strategies (169).

Interestingly, whole AV culture under flow has also been investigated in larger animal models. Konduri et al. cultured native porcine AVs for 48 h in a sterile, pulsatile organ culture system simulating physiological conditions (120/80 mmHg, 4.2 L/min). A piston pump circulated fluid through the valve, separated by a latex diaphragm to maintain sterility. The system replicated left heart function using a compliance tank, mechanical mitral valve and programmable waveform generator. Continuous monitoring of flow and pressure ensured physiological accuracy. Compared to fresh valves, cultured tissues retained ECM composition (collagen, GAGs, elastin), leaflet morphology, and cell phenotype. Endothelial integrity was preserved, and apoptosis levels remained low—comparable to fresh tissue and significantly lower than in static culture—highlighting the crucial role of mechanical stimulation in maintaining valve viability (170). Whole AV culture under flow was also assessed by Niazy et al. using ovine samples. In their system, native ovine AVs were cultured for 7 days in a bioreactor providing pulsatile flow with controlled pressure, temperature, and pH (171). Flow passed from the ventricular to the aortic side. Under dynamic conditions, type I collagen expression was maintained, unlike in static culture. Interestingly, exposure to an OM induced marked calcification in the ventricularis, with smaller deposits in the fibrosa, whereas in human CAVD, calcification typically occurs on the aortic side of the leaflet, rather than in the fibrosa. This suggests that despite pulsatile flow, shear stress distribution may not fully replicate native patterns in this model. Besides, OM exposure did not alter COL1A1, ALP, or αSMA expression, indicating limited VICs phenotypic switching in this model.

## 6 Assessing mineralization

Accurate assessment of mineralization is essential for characterizing VIC and VEC osteogenic activity and evaluating the effects of therapeutic interventions. A wide range of techniques has been developed and adapted to monitor calcification across experimental settings, including cells cultured in 2D or 3D, as well as tissue explants. These methods differ in sensitivity, specificity, quantification capacity, and applicability to *in vitro* or *ex vivo* studies. An overview of the main techniques—including Von Kossa staining, Alizarin Red staining, OsteoSense, o-cresolphthalein complexone, and ⁴⁵Ca liquid scintillation counting—along with their principles, quantification potential, and limitations, is presented in Table 4.

**Table 4:** Overview of the main techniques used to detect and quantify calcification *in vitro* and *ex vivo*, with emphasis on their specificity, sensitivity, and limitations depending on the experimental model.

Measurement type	Name of the method	Principle	Use on VICs (*in vitro*)	Use on AV tissue (*ex vivo*)	Pitfalls
Non quantitative	Von Kossa	Detects anionic calcium salts, primarily calcium phosphate deposits. It works by replacing calcium ions with silver ions, which are then reduced to metallic silver under light exposure, appearing black (177)	Detection of mineralization in fixed VICs, and AV tissues; deposits can be visualized and imaged (178)		AVs, especially in mice, contain melanin-rich melanocytes whose black/brown pigmentation can mimic mineral deposits and cause false positives in Von Kossa staining (81, 179).
Semi-quantitative	Alizarin Red Staining	Binds calcium to form a bright red complex visible to the naked eye (180)	Detection of mineralization in fixed VIC monolayers. The dye can be solubilized using a buffer containing NaH_2_PO_3_ and hexadecylpyridinium chloride, followed by absorbance measurement, providing both visual and semi-quantitative assessment (98)	Allows detection of macrocalcification and nodules in fixed AV leaflets; deposits can be visualized and imaged	Alizarin Red Staining has limited sensitivity for detecting microcrystals within AV tissue
	Osteosense	Fluorescent bisphosphonate probe that binds specifically to hydroxyapatite, enabling real-time imaging of calcification (181)	Allows detection of hydroxyapatite in cell monolayers as early as 24 h after osteogenic stimulation, with signal intensity increasing over time. Quantification of the signal allows semi-quantitative analysis of mineralization (182)	Particularly suited for detecting and quantifying microcalcifications in AV leaflets, which may be challenging with Alizarin Red (182)	Osteosense cannot detect macrocrystals and nodules within AV leaflets
Quantitative	O-cresolphthalein complexone	Binds calcium under alkaline conditions, forming a purple complex measurable by spectrophotometry.	This method involves decalcifying VIC monolayers or AV tissue with HCl to extract calcium. The supernatant containing free calcium ions is then mixed with an o-cresolphthalein complexon reagent under alkaline conditions, forming a purple complex measurable by spectrophotometry. Results are expressed as micrograms of calcium per well (for cells) or nanograms per milligram of dry tissue (for valve samples) (100)		These techniques are well suited for quantifying mineralization *ex vivo* in AVs from large animals (human, porcine, etc.). However, they are not easily applicable to rat and mouse AV, as residual myocardium (rich in calcium) often remains attached to the aortic annulus, causing bias and complicating calcium measurement
	⁴⁵Ca and Liquid Scintillation Counting	⁴⁵Ca and Liquid Scintillation Counting is a technique used to measure deposition by using the radioactive calcium isotope ⁴⁵Ca	This method involves incubating tissue samples or VIC monolayers with radioactive ⁴⁵Ca, followed by washing, drying (and weighing for tissues). Calcium is then extracted by overnight incubation with HCl, and the supernatant is analyzed by liquid scintillation counting, a sensitive technique that detects radioactive decay. Results are expressed as nanomoles of calcium per well or per milligram of tissue, based on the specific activity of ⁴⁵Ca in the medium (145)		

## 7 *In vivo* modeling of CAVD

Unlike *in vitro* models, which isolate cellular mechanisms, or *ex vivo* models, which lack systemic interactions, *in vivo* studies capture the full complexity of the disease within an integrated biological system. These models, which allow the study of CAVD in the presence of key comorbidities such as hypercholesterolemia, diabetes, and CKD, are essential for developing effective therapies. They also provide insights into the progressive nature of AV fibrocalcic remodeling and pathological changes that short-term experiments may miss, and allow evaluation of the functional impact of AV dysfunction on hemodynamic parameters and ventricular remodeling and function via echocardiography. These *in vivo* models are essential for evaluating the efficacy and safety of potential therapies, offering a physiologically relevant platform for testing pharmacological and interventional strategies before clinical trials.

### 7.1 Overview of main models

Numerous animal models have been developed to replicate the pathological conditions leading to CAVD. These models account for key risk factors and comorbidities, enabling the investigation of mechanisms such as lipid metabolism, inflammation, hemodynamics, and genetic predispositions. They can be naturally occurring, genetically engineered, diet- or surgically induced, or triggered by pharmacological agents. An overview of these models and their characteristics is provided in Table 5.

**Table 5:** Overview of the main animal models currently used to investigate CAVD pathophysiology.

Pathological context	Animal model	Experimental principle	Key features of AV remodelling									
			Features of Inflammation	Lipid accumulation	Thickening/ECM remodelling	Osteogenic transition	Calcification	Hemodynamic alterations			Limitations	Ref
								Pressure gradients	Valve orifice	LV impact		
Metabolic syndrom/Atherosclerosis	New Zealand white rabbit with a diet enriched in cholesterol	AV lesions induced by severe hypercholesterolemia within 8 weeks	ND	✓	✓	✓	✓	ND	ND	ND	Increased risk of mortality due to hepatic cholesterol overload	(183, 184)
	New Zealand white rabbit with a diet enriched in cholesterol and Vitamin D2		✓	ND	ND	ND	✓	↑MaxPG	↓AVA	ND	ND	(185, 186)
	Watanabe heritable hyperlipidemic (WHHLMI) rabbit	Genetic hyperlipidemia-induced lipid accumulation and inflammation within 30 months	✓	✓	✓	✓	✓	↑MaxPG	↓AVA	ND	Risk of spontaneous myocardial infarction	(187)
	Rapacz Familial Hypercholesterolemia swine model	AV lesions induced by severe hypercholesterolemia within 1 or 2 years	✓	✓	✓	ND	–	ND	ND	ND	AV lesions develop slowly, appearing after 1–3 years.	(188)
	WT Swine on Hypercholesterolemic Diet	AV lesions induced by severe hypercholesterolemia	ND	✓	ND	ND	ND	ND	ND	ND	ND	(189)
	ApoE−/− mice under high-fat diet	AV lesions induced by severe hypercholesterolemia	✓	✓	✓	✓	✓	ND	ND	ND	ND	(190)
	LDLr−/−, ApoE−/− C3H mice	lesions induced in the aortic root by severe hypercholesterolemia	✓ (Aortic root)	✓ (Aortic root)	ND	ND	ND	ND	ND	ND	Lesions primarily develop in the aortic root rather than the valve itself	(191)
	LDLr−/− ApoB100/100 C57BL/6J mice	The mice lack the LDL receptor, impairing LDL cholesterol clearance, and express a humanized ApoB100, essential for LDL formation. This induces severe hypercholesterolemia, lipid accumulation, and AV remodeling within 6 months	✓	✓	✓	✓	✓	ND	↓cusp separation	ND	ND	(192)
	LDLr−/− ApoB100/100 C57BL/6J mice, exposed for 20 weeks to a diabetogenic, pro-calcific diet (model of type 2 diabetes)	These mice develop hyperglycemia, hyperlipidemia, obesity, and advanced atherosclerosis, ultimately leading to AV remodeling within 6 months	✓	✓	✓	ND	✓	↑MeanPG ↑Peak velocity	↓ AVA	↓EF ↓FS	ND	(193)
	LDLr−/− ApoB100/100/IGF-II transgenic C57BL/6J mice (model of type 2 diabetes)	Expression of IGF-II combined with LDLr−/− ApoB¹⁰⁰/¹⁰⁰ genotype induces features of metabolic syndrome including insulin resistance, hyperglycemia, and obesity, and promotes AV remodeling within 6 months under a diabetogenic diet.	✓	ND	ND	✓	✓	↑Peak velocity, ↑MaxPG, ↑MeanPG	↓ AVA	↑LVM ↓EF ↓ FS ↑E/E′, ↓IVRT	ND	(194)
Type 1 Diabetes (T1DM)	ApoE−/− with IP injection of streprozotocin for 5 consecutive days and kept under hyperlipemic diet for 7 days	The development of insulin-deficient diabetes and hyperlipidemia, induced by streptozotocin injections combined with a 7-day hyperlipidemic diet, promotes AV remodeling.	✓	✓	✓	✓	✓	↑Mean LVOT VTI ↑Mean velocity	↓cusp separation	ND	ND	(195)
Chronic Kidney Disease (CKD)	SD rats with 5/6th nephrectomy + high-phosphate diet	The development of secondary hyperparathyroidism promotes AV remodeling within 11–16 weeks	✓	ND	ND	ND	✓	↑Peak velocity ↑MeanPG	↓cusp separation	↓CO ↓FS ↓EF ↑E/E′ ↑LVW ↑ LV fibrosis	Myocardial infarction that may be related to myocardial ischemia occurred in 50% of the rats after 16 weeks	(196, 197)
	ApoE−/− C57BL/6J mice with 5/6th nephrectomy	The development of secondary hyperparathyroidism together with hypercholesterolemia promotes AV remodeling within 10 weeks	ND	✓	ND	ND	✓	ND	ND	ND	ND	(198–200)
	Wistar rats with CKD induced by an adenine-rich diet	Adenine supplementation causes tubular injury and interstitial nephritis, leading to CKD and secondary hyperparathyroidism, which promote AV pathological remodeling within 9 weeks	ND	ND	ND	ND	✓	↓MaxPG ↓MeanPG	–	↓EF ↓CO	Reduced MaxPG and MeanPG contrary to typical AS progression, limiting model relevance	(201)
	ApoE-/- C57BL/6J mice, 12 weeks high-fat diet + CKD induced by 12 weeks adenine diet	Adenine supplementation causes CKD and secondary hyperparathyroidism, which, together with hypercholesterolemia from the high-fat diet, drive AV lesions within 24 weeks	ND	✓	✓	ND	✓	↑Peak velocity	↓AVA	ND	High cost: combines genetic KO with two dietary interventions + Long time to develop valvular lesions: >5 months	(202)
	Aortic valve wire injury (AVWI) in C57BL/6J mice with adenine-induced CKD	Mechanical injury to the AV cusp, combined with CKD-induced hyperparathyroidism, promotes AV remodeling	✓	ND	–	ND	–	↑MeanPG ↑Peak velocity	ND	No change in EF	ND	(203)
Hypertension	Surgical aortic constriction in Wistar rats	Surgical constriction of the ascending aorta induces pressure overload, leading to left ventricular hypertrophy	ND	ND	ND	ND	ND	ND	ND	↑HR ↑LVW ↑RVW ↓IVRT ↑FS	Although used as an AS model, the supravalvular constriction targets the aorta—not the AV leaflets, which remain structurally intact	(204)
Genetic models	Dcbld2−/− C57BL/6J mice	These mice develop bicuspid AV with a 50% occurrence	ND	ND	✓	✓	✓	↑Peak velocity	ND	ND	Calcification becomes significantly more pronounced than in TAV, but only after 10–16 months.	(205, 206)
	Notch1+/− C57BL/6J mice	Notch1 normally represses Bmp2 in murine AV; haploinsufficiency lifts this inhibition, inducing AV remodeling within 10 weeks	ND	ND	ND	✓	✓	ND	ND	ND	ND	(40)
Thoracic radiation therapy	Targeted AV irradiation in ApoE−/− C57BL/6J mice	Targeted irradiation of the AV induces accelerated remodeling that mimics human delayed radiation-induced AS within 3 months	✓	ND	Cusps and aortic sinus thickening with fibrosis	ND	✓	↑Peak velocity ↑MeanPG	ND	–	No effect on left ventricular function over time	(179, 207)
Mechanical injury	Aortic valve wire injury (AVWI) in C57BL/6J mice	AV injury is induced by advancing a spring guidewire into the left ventricle via the right carotid artery under echocardiographic guidance. Functional impairment appears by 1 week, with progressive remodeling from 4 to 16 weeks	✓	ND	✓	✓	✓	↑Peak velocity	↓AVA	↑Heart weight ↓FS ↑LVDd	ND	(208)
	AVWI + vitamin D supplementation in C57BL/6J mice	AVWI combined with vitamin D supplementation exacerbates AV remodeling within 28 days	Inflammation not further increased vs. AVWI	ND	ND	↑BMP2 vs. AVWI	↑calcification vs. AVWI	↑MeanPG vs. AVWI	AVA not further decreased vs. AVWI	ND	ND	(209)
Warfarin-based models	Administration of warfarin + vitamin K1 in SD rats	Warfarin inhibits vitamin K recycling, promoting AV calcification within 28 days by inactivating MGP, a key inhibitor of valvular calcification. Vitamin K1 prevents bleeding but does not restore MGP activity.	ND	ND	ND	✓	✓	ND	ND	↓EF ↓FS ↑LVDd ↑LVDs	Calcification quantification was performed in the aortic root, not specifically in the valve cusps	(210, 211)
	Administration of warfarin + vitamin K1 in C57BL/6J mice		ND	ND	ND	✓	✓	ND	ND	ND	ND	(212)
	Administration of warfarin + vitamin K1 in DBA/2 mice		ND	ND	ND	ND	✓	↑MaxPG	ND	ND	Calcification quantification was performed in the aortic root, not specifically in the valve cusps	(213)
	Administration of warfarin + vitamin K1 in ApoE−/− C57BL/6J mice	The treatment promotes AV calcification within 8 weeks	ND	ND	ND	ND	✓	ND	ND	ND	ND	(214)

AV, aortic valve; AVA, aortic valve area; AVWI, aortic valve wire injury; ApoE−/−, apolipoprotein E knockout; BAV, bicuspid aortic valve; BMP2, bone morphogenic protein 2; CO, cardiac output; CKD, chronic kidney disease; E/E’, ratio of early mitral inflow velocity (E) to early mitral annular velocity (E′); EF, ejection fraction; FS, fractional shortening; IGF-II, Insulin-like Growth Factor II; IP injection, intraperitoneal injection; IVRT, isovolumic relaxation time; LDL, low-density lipoprotein; LDLR−/−, low-density lipoprotein receptor knockout; LV, left ventricle; LVM, left ventricular mass; LVW, left ventricular weight; LVDd, left ventricular diastolic diameter; LVDs, left ventricular systolic diameter; MaxPG, maximal transvalvular pressure gradient; MeanPG, mean transvalvular pressure gradient; Notch1+/−, heterozygous Notch1 knockout; RVW, right ventricular weight; SD, Sprague-Dawley; VTI, velocity time integral; WT, wild type.

This table summarizes key animal models employed to study CAVD. For each model, it describes the experimental principle, main features associated with valve remodeling (inflammation, lipid accumulation, leaflet thickening and fibrosis, osteogenic transition and mineralization), echocardiographic alterations, and reported experimental limitations. ✓, parameter present or increased; –, parameter unchanged or not modulated; ND, not determined or not reported.

### 7.2 Relevance of *in vivo* models

Several critical factors must be considered when using animal models, starting with species selection. Porcine models are anatomically and hemodynamically close to humans, making them highly relevant for CAVD research. However, their use entails high costs, specialized housing, and often limits sample sizes, impacting statistical power. Rodents, widely used for their availability and genetic manipulability, differ fundamentally from humans in key aspects. For example, mice have lymphocyte-dominant white blood cells, whereas humans and pigs have neutrophil-dominant profiles, influencing immune responses (215, 216). Moreover, certain human cytokines relevant to CAVD, such as IL-8 (100), are not expressed in mice or rats, limiting the translational potential of these models. Rodent models also pose technical challenges: their tiny AV yield limited tissue for molecular analyses (e.g., Western blot, PCR), and valve isolation without myocardial contamination is difficult. Nevertheless, they remain valuable for studying cardiovascular calcification *in vivo*, including through Osteosense injection to visualize global cardiovascular calcification. Regardless of species, *in vivo* studies increasingly rely on functional imaging such as echocardiography, enabling clinically translatable assessment of hemodynamic and functional parameters. This underscores the importance of interdisciplinary collaboration with clinicians and imaging specialists to ensure accurate and meaningful data interpretation. Despite significant advances, Table 5 shows that no animal model to date fully recapitulates the complex pathophysiology of CAVD—including fibrosis, lipid accumulation, calcification, elevated transvalvular gradients, valve narrowing, and left ventricular remodeling. As a result, we still lack a reliable preclinical model for testing therapeutic interventions aimed at preventing the onset or halting the progression of AS, despite considerable research efforts in this field. Developing such a model remains a critical need to improve our understanding of disease mechanisms and to enable robust therapeutic screening. To develop reliable models of valvular calcification, critical details—such as animal strain, sex, and diet composition—should be systematically reported in every published study. To this end, researchers are encouraged to follow the ARRIVE guidelines, which provide a comprehensive checklist of essential information for *in vivo* studies. Adherence to these guidelines allows evaluation of methodological rigor, facilitates experiment reproducibility, and ensures accurate interpretation of results. It also promotes complete reporting of experimental design, randomization, blinding, sample size justification, and outcome measures, thereby enhancing study quality and transparency (217). A more standardized approach will not only enhance reporting consistency but also aid in selecting appropriate methodologies, ultimately accelerating the identification of suitable animal models for CAVD.

## 8 Conclusion

**Figure 3:**
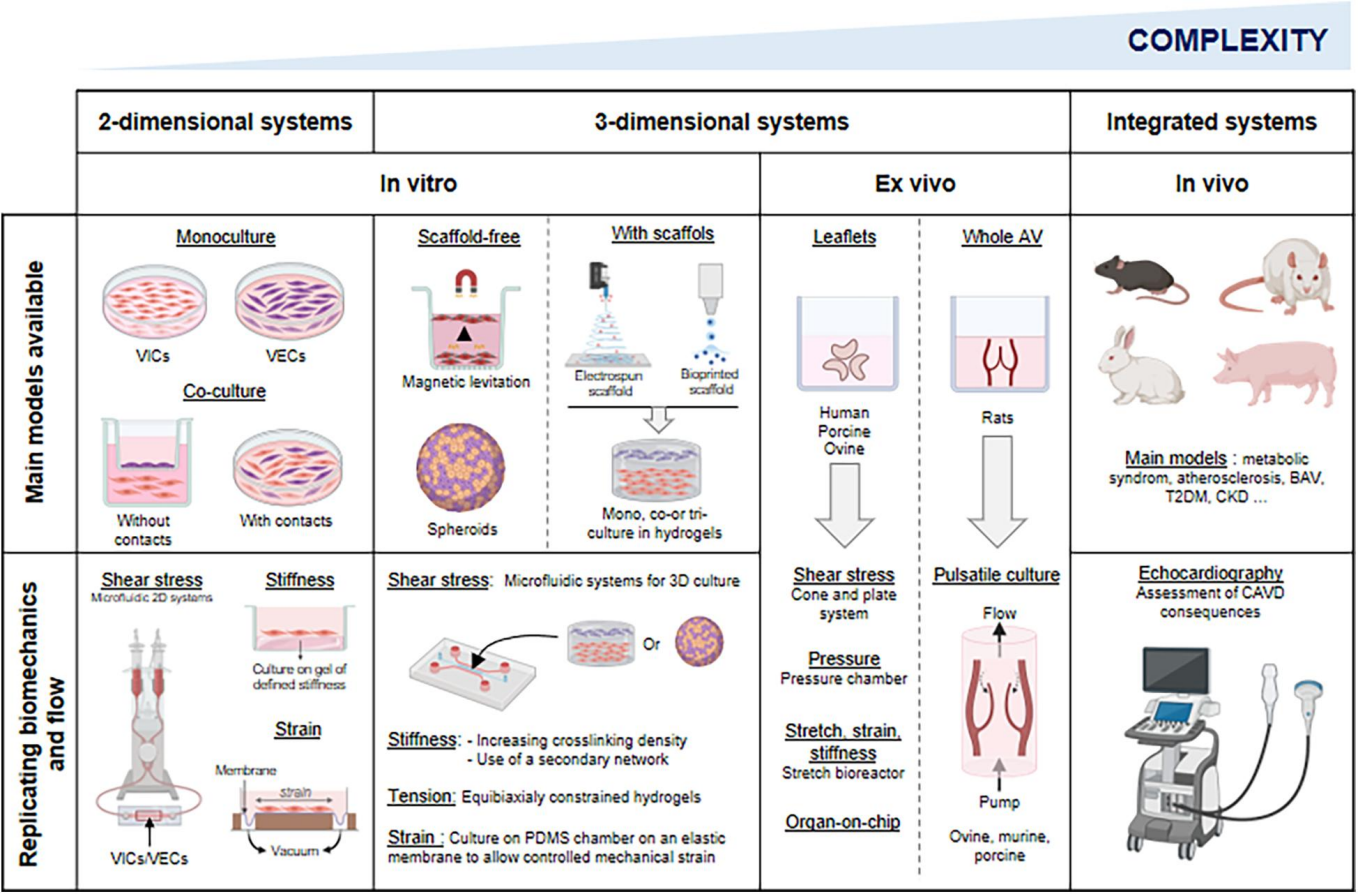
Overview of the main models currently available to study CAVD. In this figure, models are organized by increasing complexity and physiological relevance from left to right. The upper row illustrates static models, where mechanical and hemodynamic stimuli are absent. The lower row presents dynamic models, incorporating mechanical forces and flow conditions to better mimic the physiological environment of the aortic valve. This figure highlights the progressive refinement of experimental systems, from simple 2D *in vitro* cultures to complex *in vivo* models, to guide model selection based on research objectives. Illustration created with BioRender.

Although no model fully replicates the native AV environment and the mechanisms driving CAVD, significant efforts have been made to develop robust experimental systems (Figure 3). Two-dimensional *in vitro* models offer precise control of the cellular environment, making them useful for studying signaling pathways and cell–cell interactions. However, they fail to mimic the native valve's 3D architecture and cell–matrix interactions. Three-dimensional models—based on aggregates, scaffolds, leaflet fragments, or whole aortic valves—better address these limitations. The incorporation of hemodynamic flow and mechanical forces into these *in vitro* and *ex vivo* systems represents a major advance. Despite these improvements, *in vivo* models remain essential for studying CAVD progression within an integrated biological context. In this regard, the development of a zebrafish model, which would enable high-throughput compound screening, is highly anticipated (218, 219).

When modeling CAVD, it is important to keep in mind that each model provides only a partial perspective on disease mechanisms; thus, combining complementary approaches is often the most effective strategy for addressing complex research questions. However, the multifactorial nature of CAVD, which often requires a focus on specific risk factors, continues to limit the generalizability of findings and remains a major obstacle to fully replicate the disease. Among these factors, BAV represents a major risk for early-onset and accelerated CAVD. Yet, few preclinical systems have been specifically developed to modelize this morphological variant. Indeed, the vast majority of existing models rely on tricuspid valve anatomy, limiting their ability to reflect the unique hemodynamic and mechanical conditions associated with BAV. This remains an important gap in the field and underscores the need for future models tailored to this clinically significant phenotype. In the future, integrating spatial transcriptomics, scRNA-seq, and other high-resolution techniques into studies of bicuspid and tricuspid valves, as well as key comorbidities, is expected to enhance our understanding of the mechanisms driving AS. The standardization of trilayered in-flow organoid systems and AV-on-chip platforms still remain key challenges for the coming years and could significantly enhance translational potential.
